# Beyond Species Diversity: Functional Approaches Reveal Consistent Fauna Community Responses to Exotic Grass Invasion in Arid Lands

**DOI:** 10.1002/ece3.73334

**Published:** 2026-04-09

**Authors:** Ellen Ryan‐Colton, Glenda M. Wardle, John L. Read, Kris French, Christine A. Schlesinger

**Affiliations:** ^1^ Research Institute for the Environment and Livelihoods Charles Darwin University Alice Springs Northern Territory Australia; ^2^ School of Life and Environmental Sciences The University of Sydney Sydney New South Wales Australia; ^3^ College of Science Adelaide University Adelaide South Australia Australia; ^4^ School of Science University of Wollongong Wollongong New South Wales Australia

**Keywords:** biological invasions, buffelgrass, functional framework, multi‐taxa, trait‐filtering

## Abstract

Fauna communities respond to exotic plant invasions through multiple pathways, including via changes to habitat structure and food resources and depending on the scale at which fauna access these resources. To assess whether such multi‐dimensional impacts on fauna can be generalised across taxa, we developed and empirically tested a conceptual framework to predict how fauna communities respond to exotic grass invasion in open arid ecosystems. We predicted the greatest impact on combinations of lower trophic levels or diet‐specialists, ground‐active fauna, including open habitat specialists, and species that operate over smaller spatial scales. We further proposed that functional analysis would more readily detect impacts than total taxon abundance, diversity or taxonomic composition. Replicated across two regions of arid central Australia, we sampled birds, reptiles and ants at native sites and paired sites invaded by exotic buffel grass ( *Cenchrus ciliaris* ). Results largely validated our predictions. Bird and ant communities showed significant functional homogenisation or restructuring at invaded sites, respectively (PERMDISP or PERMANOVA *p* ≤ 0.005), with some functional differences also evident for reptiles (*p* = 0.02), despite low captures of this group with dry conditions. Ground‐active reptiles, birds and ecologically dominant ant groups, especially those that use open microhabitats, were less characteristic of invaded sites. Diet specialists were less associated with invaded sites, including insectivorous birds, and granivorous ants and birds, except where granivores operated at landscape scales. Whilst ant abundance was reduced by 50% (0.41 [0.25–0.68, 95% CI]) and bird communities showed taxonomic homogenisation in invaded sites (PERMDISP *p* = 0.005), no impacts on taxonomic diversity were detected. Functional responses provided the clearest and most consistent detection of community‐level impacts in a multi‐taxa context. This validates key aspects of our conceptual framework and offers a robust, transferrable approach for analysing exotic grass invasions and other drivers of ecological change.

## Graphical abstract

To assess whether the impacts of exotic plant invasion can be generalised across taxa, we developed a conceptual framework predicting faunal responses to grass invasion in open, arid ecosystems and tested our predictions using data on birds, reptiles and ants from sites invaded by buffel grass ( *Cenchrus ciliaris* ) and paired uninvaded sites. Diet specialists and ground‐active fauna were generally less associated with invaded sites, aligning with our predictions. Functional responses provided the most consistent signal of community‐level impacts of invasion on fauna, more so than abundance or diversity metrics. With validation of key aspects of our framework, this multi‐taxa and multi‐trait approach could be used for other invasions and environmental change.

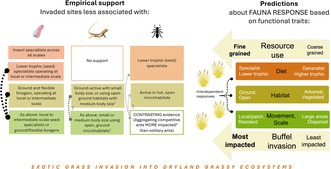

## 1 Introduction

Exotic grasses are serious invaders worldwide (D'Antonio and Vitousek 1992; Williams and Baruch 2000; van Klinken and Friedel 2017) and are expected to have a major and increasing impact on fauna as they modify vegetation structure and diversity (Steidl et al. 2013) and alter ecosystem processes at local and continental scales (Gaertner et al. 2014; Fusco et al. 2019). However, meta‐reviews of invasion impacts have yet to detect consistent impacts on fauna in semi‐arid and arid regions or in grasslands at a global scale (McCary et al. 2016; Fletcher et al. 2019). Inconsistent faunal responses to invasive grasses are not surprising, considering the varied dryland systems that have been invaded and the multiple direct and indirect mechanisms by which invasive grasses alter habitat structure, food resources and species interactions (Steidl et al. 2013; Bezemer et al. 2014; Litt and Pearson 2022). Even within a particular ecosystem, disparate responses among species make predictions about impacts at the community level difficult (Kutt and Fisher 2011; Litt and Steidl 2011). The rapid global expansion of exotic plants globally (Seebens et al. 2025) demands approaches that can identify generalisable responses of fauna to environmental change (Wardle et al. 2013) to swiftly predict and prioritise species and ecosystem functions most at risk from plant invasion.

To discern general trends, one approach is to examine common responses to habitat change among functional guilds of species that share traits or ecological roles (A. Andersen 1995; French and Zubovic 1997; Tischler et al. 2013; Santos et al. 2021). Functional or trait‐based approaches can reveal community‐level change that is not apparent from total abundance or diversity alone (Litt and Steidl 2011; Read et al. 2015). Consistent functional responses across taxa or trophic levels indicate that ecological drivers act broadly across the community, strengthening mechanistic inference and improving the capacity to predict ecosystem‐level responses to further disturbance. However, multi‐taxa and functional approaches have been underutilised in invasion ecology (Fletcher et al. 2019) and community ecology more broadly (Seibold et al. 2018).

**FIGURE 1:**
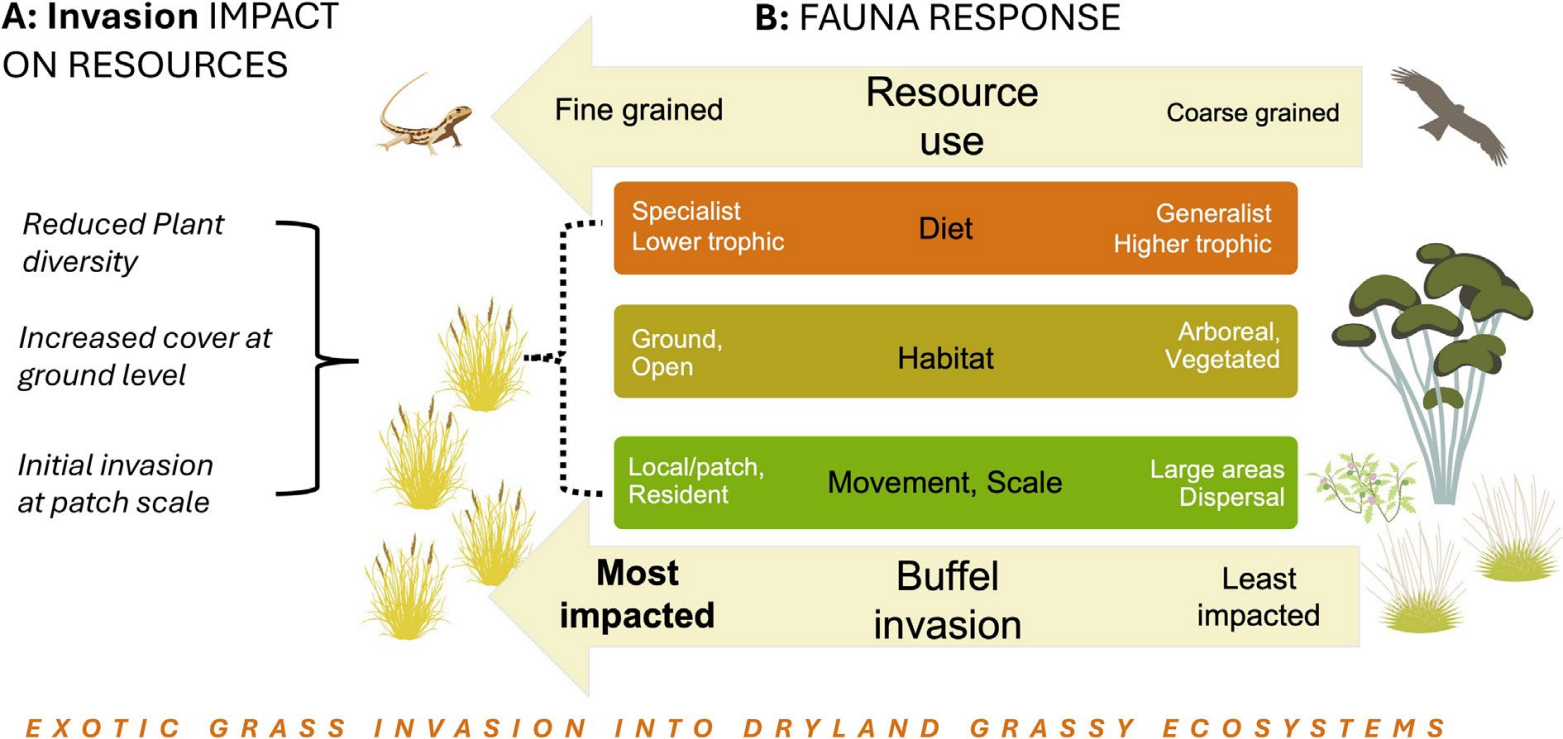
Conceptual model for the functional responses of fauna to grass invasion, in arid grassy ecosystems. (A) Key biotic and structural changes in the plant community predicted to impact fauna. (B) Diet (food) and habitat are essential for all faunal species while movement patterns and spatial scale determine access to these resources. Our model predicts the greatest impacts on fauna with more specialised or fine‐grained resource use in relation to the specific known impacts of invasion (A) including fauna with more specialised or lower trophic level diets, terrestrial fauna that make extensive use of open habitat with bare ground; and species operating at smaller scales with limited opportunity to move away from invaded patches. These functional responses are expected to be interdependent (dashed line), reflecting the complexity of fauna response to invasion. Fauna symbols provided by Indigenous Desert Alliance, here and throughout.

To address this gap, we first devised a conceptual model to formulate testable predictions about functional responses of faunal communities to grass invasion, focusing on the transition from open native grasslands to dense perennial grasslands in hot, arid climates (Figure 1). Our conceptual model focuses on the ecological changes associated with buffel grass ( *Cenchrus ciliaris* L. syn *Pennisetum ciliare* (L.) Link.), a widespread exemplar of grass invasions in dry ecosystems, from the arid zone to dry subtropical biomes. Buffel grass has displaced native plant communities across millions of hectares in Australia, North, Central and South America and the Pacific (Marshall et al. 2012) and shares many characteristics with other pervasive exotic grasses (Williams and Baruch 2000). Notably, buffel grass is a deep‐rooted perennial C4 grass; buffel grass exhibits high tolerance to drought, promotes and is promoted by fire, has taller stature and faster biomass accumulation than resident natives, and produces prolific seeds (Miller et al. 2010; Marshall et al. 2012; Schlesinger et al. 2013; Kumar et al. 2019; Schlesinger and Westerhuis 2021).

Central to our model are three previously well‐established outcomes of buffel grass invasion (Figure 1): a decline in plant diversity; an increase in ground vegetative cover; and progressive invasion from patch to larger scales (Clarke et al. 2005; Olsson, Betancourt, Crimmins, and Marsh 2012; Olsson, Betancourt, McClaran, and Marsh 2012; Schlesinger et al. 2013, 2020; Ryan‐Colton et al. 2024). We predicted how fauna would respond to these habitat and resource shifts using three key functional traits—diet (breadth and trophic level), use of habitat (ground, open, arboreal) and scale of resource use (local patch, widespread). We selected these traits to reflect contrasting dimensions of species' sensitivity to invasion (Figure 1).

Lower trophic levels, that is, plant‐consumers, tend to show more conspicuous change with plant invasion (McCary et al. 2016), compared to higher trophic levels (Fletcher et al. 2019) because they are impacted more directly. Specialist fauna are expected to be replaced by generalist fauna, given generalists can more readily adapt to habitat change, and specialised niches may be lost with resource homogenisation (Clavel et al. 2011). Importantly, different combinations of traits may either mitigate or concentrate impact. For example, because native seed resources are often significantly reduced following invasion (Wright et al. 2020), and granivores that specialise on native seeds are expected to be particularly impacted (Young and Schlesinger 2018), such impacts may be especially severe for species that operate on small scales and are unable to access uninvaded areas. Mobile faunal groups can potentially move between invaded and uninvaded patches and negate resource‐depletion (or structural change) at the patch scale (Figure 1), at least in early stages of invasion (Martin and Murray 2011). However, when invasion occurs over large areas and multiple habitat types, even highly mobile fauna may be impacted as patches of preferred habitat are altered.

Habitat structure, particularly the openness of habitats, has a major influence on the composition of arid zone communities (e.g., ants: A. N. Andersen 2019, birds: MacArthur and MacArthur 1961, reptiles: Pianka 1966). Many arid‐adapted species that rely on areas of bare ground for efficient locomotion, foraging or thermoregulation (Pianka 1986; Read 1995; Kutt and Fisher 2011; Steidl et al. 2013) are likely to be substantively impacted by increases in ground‐level grass cover, consistent with documented community‐level shifts under invasive grass expansion (Smyth et al. 2009; Bonney et al. 2016). In contrast, fauna that use arboreal habitats are expected to be comparatively less impacted in the early stages of invasion (Young and Schlesinger 2014; Neilly et al. 2017; Marino et al. 2022), when displacement of understorey vegetation is the primary change to habitat (Figure 1).

Existing trait‐based frameworks for fauna responses to disturbance or invasion are generally limited to single taxonomic groups (e.g., reptiles: Martin and Murray 2011; ants: A. N. Andersen 2019). Some global‐scale multi‐taxa syntheses incorporate fauna traits (McCary et al. 2016; Fletcher et al. 2019; Marino et al. 2022), but do not consider how plant invasion alters key ecosystem traits that subsequently shape faunal responses. Litt and Pearson's (2022) framework integrates changes along the resource axis but does not quantitively account for multidimensional or combined effects which may be particularly important in drylands where resource availability is spatially and temporally limited. Our framework addresses these gaps by explicitly linking invasion‐driven ecosystem changes with multidimensional trait combinations across both invertebrates and vertebrates, within a clearly defined invasion context relevant to arid grassland systems.

We aimed to test the predictions of our conceptual model by investigating the impacts of buffel grass invasion on three major faunal groups: birds, reptiles and ants. Specifically, we predicted that (1) the functional group structure of communities would differ between invaded and native sites, with the strongest differences occurring for fauna with combinations of specific traits, namely: lower or specialised trophic diets, terrestrial species reliant on open habitats, and taxa operating at smaller scales; and (2) measures of total abundance, diversity or taxonomic composition would be less effective than functional groups in detecting invasion impacts across taxa.

## 2 Materials and Methods

### 2.1 Study Area

**FIGURE 2:**
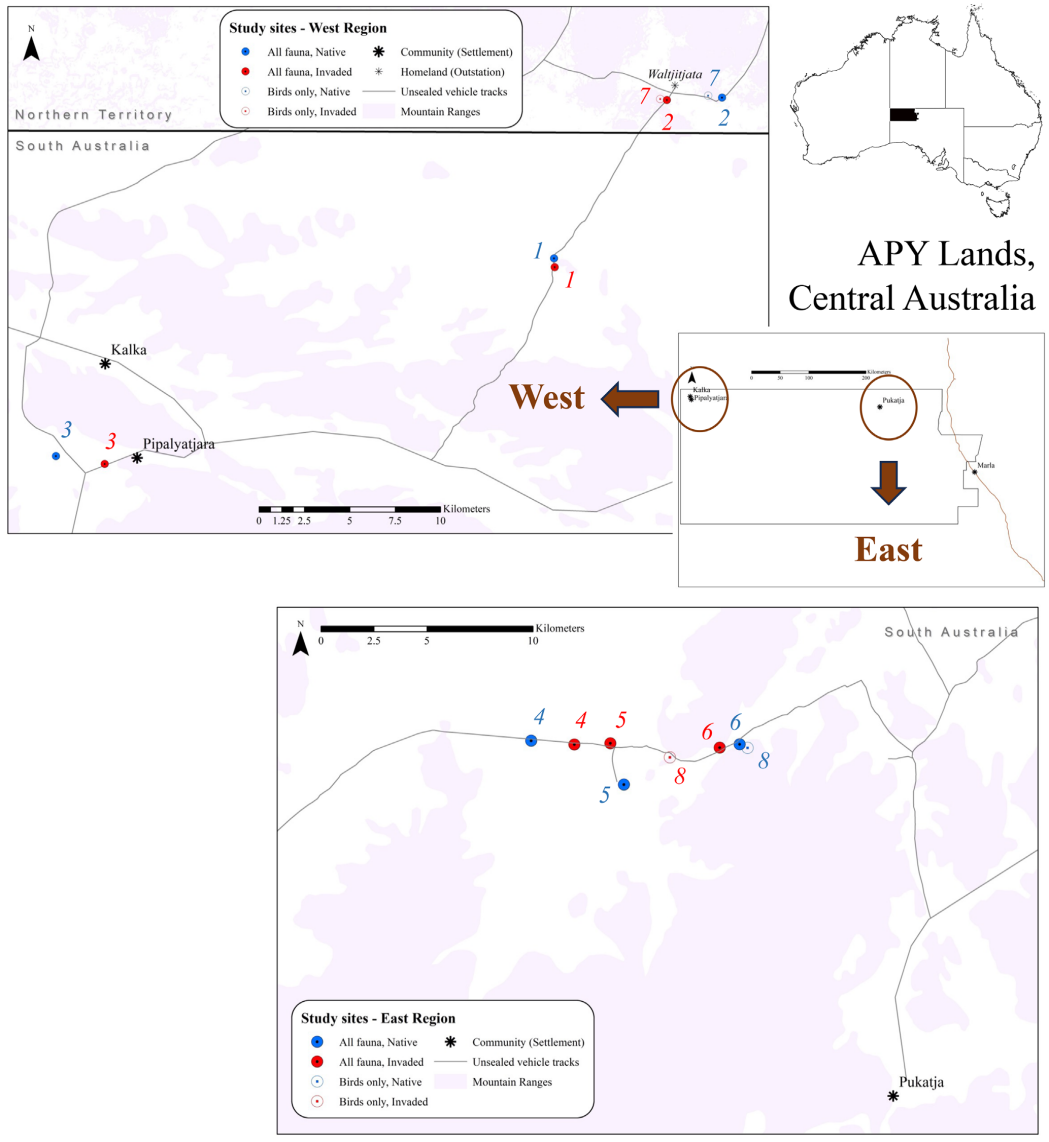
Site locations in the West and East regions of the Aṉangu Pitjantjatjara Yankunytjatjara (APY) Lands, central Australia, separated by ~300 km. Sites are numbered as pairs (1–8), with each site pair reflecting the same plant community, with native (blue) and invaded (red) sites in a randomised‐block design. Birds were surveyed at all 16 sites including rocky hills (mountain ranges in purple), while reptiles and ants were surveyed at 12 sites (pairs 1–6) sandy to alluvial plains (shown in white). The West region was surveyed in September 2018, and the East in October 2019.

The study was conducted in two regions within the Aṉangu Pitjantjatjara Yankunytjatjara (APY) Lands in central north‐west South Australia, approximately 300 km apart: the West region (−26.1380 latitude, 129.1620 longitude) near Kalka‐Pipalyatjara settlement and the East region (−26.2794, 132.1341) near Pukatja (Ernabella) settlement (Figure 2). The study region, habitat types and history of buffel grass invasion is described in more detail in Robinson et al. (2003) and Ryan‐Colton et al. (2024). Key environmental aspects relevant to fauna include a hot and arid climate, experiencing highly irregular rainfall (average 260 mm per year) and extreme seasonal temperatures, ranging from an average maximum of 20°C in winter and 40°C in summer (Bureau of Meteorology 2021). The region is characterised by rocky mountain ranges with surrounding flatter sand or alluvial plains, with plant communities generally open in structure, dominated by overstorey *Acacia, Senna* and/or *Hakea* shrubs and trees and understorey tussock grasses (*Aristida, Digitaria*, *Enneapogon, Eragrostis* spp.) or spinifex hummock grasses (*Triodia* spp.).

### 2.2 Experimental Design

**FIGURE 3:**
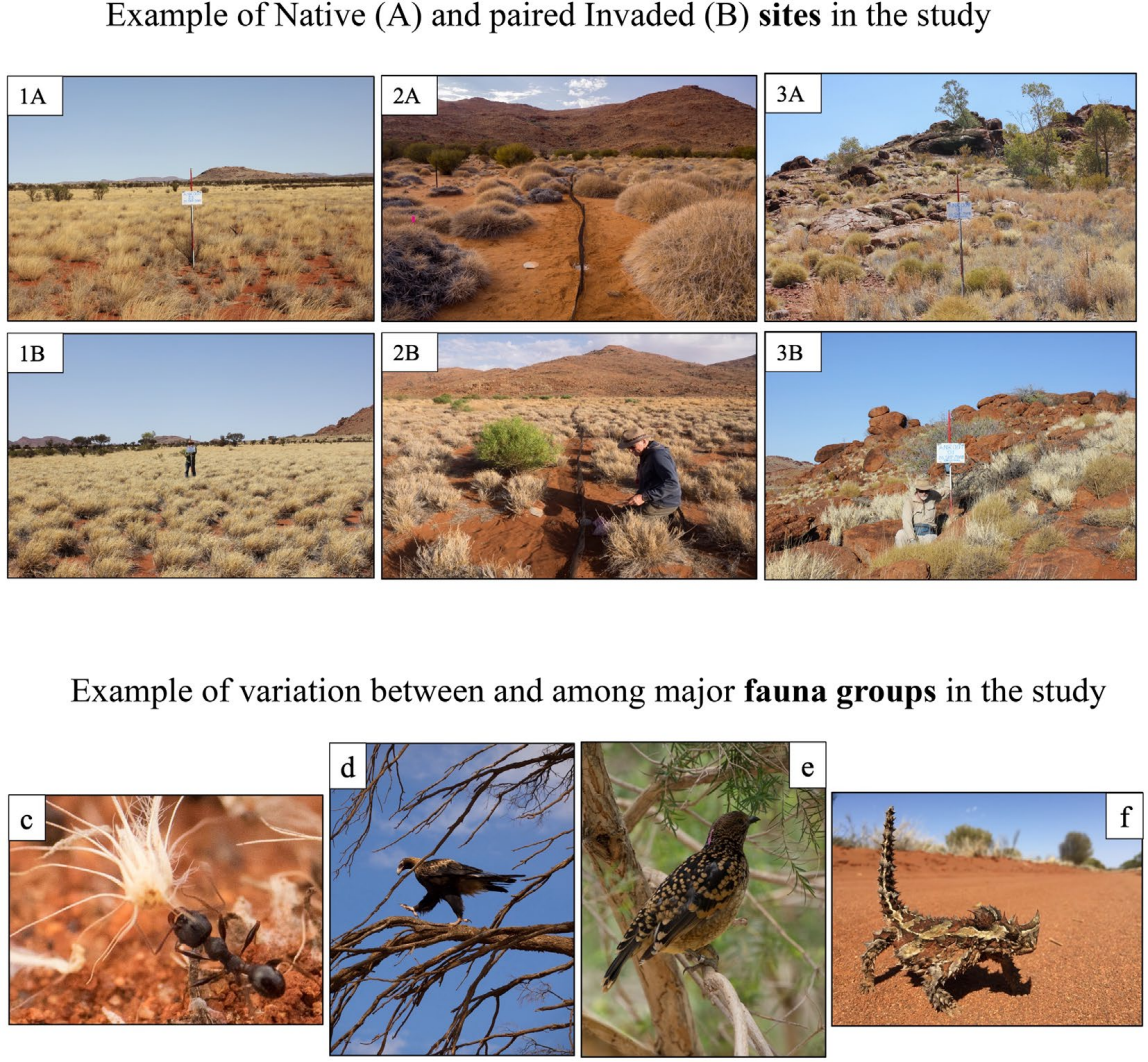
Examples of different arid zone vegetation communities (examples 1, 2, 3) that were used to test invasion effects, with each community having a native (A) and buffel grass invaded site (B), with the same landform, soil type and overstorey structure (if present). Species were grouped according to their function to enable comparisons across major Australian arid zone taxa (ants, birds, reptiles), highlighting the contrasting use of habitat structure, mobility/home range, trophic level and diet specialisation, within (d, e) and between (c–f) taxonomic groups. Photos: D. Bickerton, P. Lang, P. Canty (see acknowledgments) and E. Ryan‐Colton.

Fauna communities were surveyed via a paired‐plot (randomised‐block) experimental design primarily on plains (all taxa, 12 sites) but also rocky hills (birds only, 4 additional sites) across the West and East region (Figure 2). Thus, the design comprised 12 sites for reptiles and ants and 16 sites for birds. Sites were habitat patches of uniform vegetation, of at least 0.1 km^2^ (e.g., 200 m × 500 m), and sampling within each patch was at scales appropriate to each taxonomic group (Seibold et al. 2018, further described below). Sites were paired, representing the same plant community (blocking factor), but with one site in native condition and one site invaded by buffel grass. Site pairing kept habitat variables of soil type, landscape position (aspect) and, if present, overstorey vegetation structure constant (Figure 3), which was assessed by vegetation surveys. Native sites were defined as those with 0%–5% cover of buffel grass, and invaded sites with buffel grass cover > 5%, typically in the range of 10%–60%. Invaded and native sites within each pair were separated by one to three kilometres, and site pairs were interspersed across the landscape and evenly split across regions (Figure 2). Surveys in each region were undertaken a year apart: with the survey in September 2018 (West region) following drier than average climatic conditions, and the survey in October 2019 (East region) occurring during the driest year on record (Bureau of Meteorology 2021). Thus, *Region* as a factor in the experimental design accounts for both differences in fauna communities based on geography and seasonal conditions leading up to the surveys. The full experimental model included *Invasion status* (Native vs. Invaded, fixed) and *Site pair* (blocking factor, random) nested within *Region* (West vs. East, fixed): Response variable ~ Invasion status (fixed) * Region (fixed) + (1|Site pair).

### 2.3 Field Surveys

#### 2.3.1 Site Layout

Birds, reptiles, ants and vegetation were surveyed concurrently at each site over 4 days in September 2018 (West region) and 4 days in October 2019 (East region). Within each uniform habitat patch (> 0.1 km^2^), vegetation was sampled within a one‐hectare plot (100 m × 100 m) at the centre of each site. Reptiles and ants were surveyed along two 60 m traplines, one within and one outside the one‐hectare plot within the extended habitat patch. Bird surveys were conducted over the one‐hectare plot and extended habitat patch, covering between 0.1 and 0.15 km^2^ in a timed survey.

#### 2.3.2 Vegetation and Habitat Variables

**FIGURE A1:**
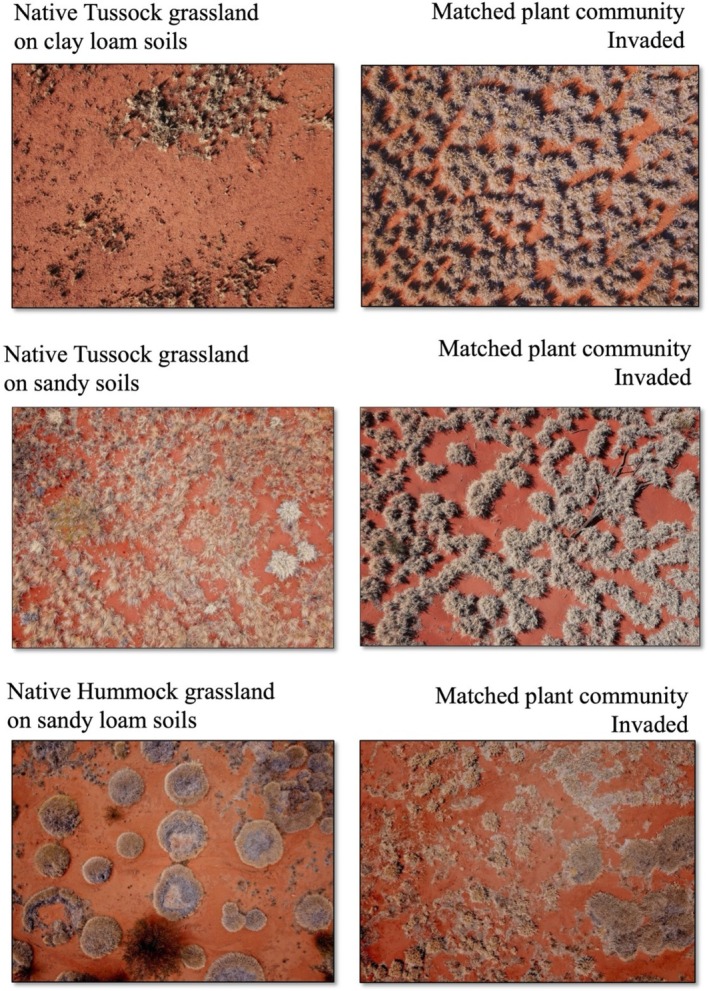
Examples of three grass‐dominated plant communities in the paired‐plot design (out of eight plant communities total). The communities are: (top left) native tussock grassland on heavier soils (*Enneapogon avenaceus, Aristida latifolia, Astrebla pectinata*); (middle left) native tussock grasslands on sandier soils (*Aristida holathera var. holathera*); and (bottom left) native spinifex hummock grassland on sandy loam soils (*Triodia irritan*s). Corresponding plant communities invaded by buffel grass are shown on the right. To aid visual comparisons (middle left) buffel grass is starting to invade native *A. holathera* grassland, and (bottom right) some mature native *T. irritans* hummocks remain unburnt surrounded by buffel grass that invaded after a fire three years prior. Imagery taken at 30 metres altitude using MavicPro drone by author ERC, which are examples of top‐down images used to estimate percent cover via image analysis.

Vegetation sampling methods followed Heard and Channon (1997) and are reported in more detail in Ryan‐Colton et al. (2024). At each one‐hectare plot we estimated plant species cover abundance and estimated the percent cover of bare ground and litter. We then calculated the relative cover of six habitat variables: buffel grass, bare ground, litter, native understorey plants, overstorey shrubs/trees (dead or alive) and coarse woody debris. On the flatter plains (12 all taxa sites) percent cover was estimated using point‐sampling image analysis (SamplePoint, Booth et al. 2006) from top‐down drone images (Mavic Pro, e.g., Appendix A: Figure A1). Steep inclines on rocky sites led to oblique drone images and thus image analysis was not suitable for these sites (which were only surveyed for birds), so for consistency for bird sites, the ground‐based visual estimates of percent cover for the habitat variables were used for all bird sites.

#### 2.3.3 Fauna

Fauna sampling methods followed Owens (2000). Each of the 16 bird sites was surveyed twice over a 4‐day period, once in the morning (between 0600 and 1000) and once in the afternoon (1400–1900), totalling 32 surveys. Each survey was a one‐hour area search, with observers walking through the vegetation plot and extended habitat patch. All birds seen or heard were recorded, including the number of individuals, which was scored as ‘1’ for birds that were only heard. Bird surveys were conducted by experienced observers, PB, PCo, JD, JH and LI (see acknowledgements), and CS and no site was resurveyed by the same observer.

Reptiles (12 sites) were surveyed along two trap‐lines per site separated by a minimum of 75 m and a maximum of 300 m. Each trapline comprised 6 pitfall traps, spaced 10 m apart, connected with a flywire drift fence (300 mm high). Pitfall traps were 140 mm in diameter and depth 380 mm, made from rolled poly‐plastic sheet (455 mm × 380 mm) held together with a plastic connecting strip and a metal flywire bottom held in place with a rubber band (Owens 2000). Traps were set for 4 consecutive nights and checked early morning and late afternoon. Captured reptiles were identified in the hand to species, individually marked (texta on ventral side) so that recaptures could be identified, and then released immediately on site. Reptiles were also recorded if they were observed within 10 m either side of the trapline whilst checking the traps. Reptile taxonomy followed Hutchinson (2023).

Ants (12 sites) were surveyed using two trap‐lines per site with six micro‐pits per line as for reptiles, totalling 12 micro‐pits per site. Each micro‐pit was placed approximately five m away from each reptile pitfall trap. Micro‐pits were 40 mL plastic vials (80 mm height × 25 mm diameter) buried into the substrate with the open top flush with the ground and filled fully with ethanol (70% or 100%). Traps were left open for 4 consecutive nights and ethanol was topped up daily or as needed if partly evaporated. Traps were removed and sealed with lids after four nights with specimens and ethanol inside and transported off‐site for identification. We also collected ants from the reptile pitfall trap buckets daily (as per Andersen et al. 2002) and stored them in a separate ethanol vial. Ant specimens in each vial were identified and counted post‐survey by ERC (author) and JW (in acknowledgements), with taxonomy to genus. Specimens were lodged with the South Australian Museum.

### 2.4 Classification of Fauna Functional Groups

Functional groups for birds, reptiles and ants were defined to best represent three functional axes of (1) diet, (2) habitat use and (3) the scale of resource use in a way that was meaningful for each taxon (Appendix A: Tables A3, A8, A12). For example, the scale of resource use was defined by proxies of movement patterns (birds), body size (reptiles) or aggregation size (ants) to ensure taxa within each group could be represented along this axis, and we acknowledge these three taxa operate at different scales to begin with.

Birds were classified into five *diet* groups to reflect trophic and specialisation level—Granivore, Nectar/Frugivore, Omnivore, Insectivore, Carnivore—based on the most common dietary classifications from four studies of arid bird communities (Smyth et al. 2009; Tischler et al. 2013; Jordan et al. 2017; Gorta et al. 2022) and with reference to Garnett et al. (2015). Birds were also classified based on their use of *vertical strata for foraging*—Ground, Above Ground or Flexible (uses both)—following classifications in two previous studies of buffel grass impacts on birds (Smyth et al. 2009; Young and Schlesinger 2014). Birds were also classified into three groups to represent their *movement patterns*: (1) Local scale, for birds that are persistent through time and resident; (2) Local‐Landscape scale, that are either variable through time or nomadic/migratory, but not both, and (3) Landscape scale, that are both variable through time and nomadic/migratory. For this we consulted Gibson et al.'s (2022) classification of arid zone birds, supplemented with Garnett et al. (2015), which contained population trend data to distinguish persistent from variable, and dispersal traits to define resident (local dispersal only) or nomadic/migratory (complete or partial migrants or nomadic, irruptive or opportunistic dispersal).

Reptiles were classified into four *diet* groups to reflect trophic level and distinguish specialists from generalists—Invertebrate specialists, Invertebrate generalists, Vertebrate specialists and Vertebrate generalists—based on Pianka (1986), Letnic et al. (2004) and supplementary literature (Appendix A: Table A8). Invertebrate specialists included ant and termite specialists, whereas Invertebrate generalists included reptiles with diets that include a range of invertebrates or with omnivorous diets, which is occasionally reported in the literature (e.g., *Ctenophorus nuchalis* consumes some plant matter Daly et al. 2008, and *Gehyra vesicolor* can digest tree sap, Edwards et al. 2021). Vertebrate specialists included species that primarily feed on squamate eggs only, whereas Vertebrate generalists included consumption of varied vertebrate animals. Where no published literature was available for a species, information from congenerics was used. Reptile *habitat use* was defined as a combination of preference for open or vegetated habitats, and vertical habitat use (subterranean, ground level or arboreal) resulting in five categories—Open at Ground level, Vegetated‐Open (uses both) at Ground level, Vegetated at Ground level, Arboreal and Subterranean. Habitat use was interpreted from space–time partitioning in Pianka (1986), supplemented with other literature (Appendix A: Table A8). Where no habitat use information was available, we used Letnic et al. (2004) post‐fire age groups as a surrogate for open to vegetated ground level habitat use. Reptiles were also classified according to *body size* as a proxy for home range size, to represent scale of resource use (Perry and Garland 2002; Todd and Nowakowski 2020). This is distinct from the use of body size to delineate reproductive strategy as per other studies (Marino et al. 2022). Average body size for each species derived from Wilson and Swan (2013) (snout vent length for lizards, total length for snakes) was plotted in a frequency plot to identify four body size clusters: 30‐80 mm (millimetres), 90–140 mm, 220–330 mm, 350–800 mm (Appendix A: Table A8).

Given that fauna can respond to invasion via multiple traits that may be interdependent, for birds and reptiles we coalesced the three functional axes (diet, habitat use and scale) into two‐way combinations, such that final functional groups for each taxon represented their diet × habitat use, diet × scale and habitat use × scale. For example, a bird species classified as a Granivore, Ground level forager at the Local scale was classified into the two‐way combinations of Granivore.Local, Granivore.Ground Level and Ground Level. Local, and these two‐way groups were used in the multivariate analysis. Two‐way combinations were chosen over three‐way due to the sparse data collected in the dry sampling period, as three‐way combinations would have been akin to analysing by species.

Ant functional groups have been well described in the context of predicting their response to environmental change (A. Andersen 1997; Santos et al. 2021), and these pre‐defined groups were used. They included: Dominant Dolichoderinae (DD), Hot Climate Specialists (HCS), Generalised Myrmicinae (GM), Opportunists (OPP), Subordinate Camponoti (SC), Cryptic species (CRY), Specialist Predators (SP), Cold Climate Specialists (CCS) (Appendix A: Table A12). Analysis was performed on these groups, and subsequently interpreted post hoc along three functional axes. Ant functional groups were interpreted based on *temperature tolerance*—Hot, Broad or Cool—to reflect their preferences for open vs. shaded microhabitats (A. Andersen 1995, 2019; Santos et al. 2021). Groups were also interpreted according to *diet*, based on trophic level and whether they included specialist seed harvesters. Our levels were defined—Includes specialist seed harvesters, Omnivores—plant dominant, Omnivores—animal dominant and Predators (Briese and Macauley 1981; A. Andersen 1997). Finally, groups were examined by different levels of *hierarchy—*Dominant, Competitive but avoids Dominant and Poor competitors (A. Andersen 1995), whereby dominant and competitive groups can form large aggregations, but poor competitors generally do not (Andersen and Patel 1994; A. Andersen 1995; Gibb et al. 2023). Increasing hierarchy and aggregation size was used as a proxy for scale of resource use (Gibb et al. 2023), as we reasoned that: (a) larger aggregation sizes equated to a larger organism (eusocial ant colony) with numerous workers foraging, leading to (b) larger area of resource use compared to a solitary ant when considering all workers (even though solitary individual may still forage widely) and (c) being more competitive would facilitate resource monopoly, effectively having access to more resources than poor competitors (Lach et al. 2009; Gibb et al. 2023).

### 2.5 Data Analysis

To test for community‐level differences associated with buffel grass invasion, we used PERMANOVA to assess multivariate community composition and PERMDISP to assess the variance (dispersion) of community composition between invaded and native sites. Analyses were conducted in PRIMER‐e with PERMANOVA add‐on (Clarke and Gorley 2006; Anderson et al. 2008).

PERMDISP calculates the average distance of samples within a group to that group's centroid in multivariate space (Anderson et al. 2008). Smaller dispersion indicates that communities are more similar to one another in their species or functional composition, whereas larger dispersion reflects greater among‐site variability. Reduced dispersion is therefore interpreted as functional or taxonomic homogenisation, that is a loss of distinct community components unique to each site (Olden and Poff 2003). PERMANOVA detects differences in the location of group centroids and their variance (dispersion), which is interpreted as an overall difference in community composition.

Both analyses used percentage difference (Bray–Curtis) dissimilarity matrices, Type III sums of squares and unrestricted permutations of the raw data, an approach appropriate for small sample sizes (Anderson et al. 2008). PERMANOVA tested for an effect of invasion depending on region using the full experimental model *Invasion status × Region + (1|Site Pair)*, and PERMDISP examined the main effects including the interaction term. Significance of effects and interactions was assessed using permutation‐based *p* values for both PERMANOVA and PERMDISP.

Univariate responses (abundance and diversity) were analysed using mixed models in RStudio (R Core Team 2023), following protocols in Ryan‐Colton et al. (2024). We used the full experimental model *Invasion status × Region + (1|Site Pair)*, and subsequently tested reduced models (*Invasion status + Region + (1|Site Pair)* or *Invasion status + (1|Site Pair)*). The most parsimonious model was selected using model diagnostics (DHARMa; Hartig 2021) and lowest AICc (bbmle; Bolker et al. 2021). Effects were considered significant when 95% confidence intervals of model estimates did not overlap zero (or one for generalised models), supported by likelihood ratio tests.

#### 2.5.1 Detecting Impacts of Invasion Using Total Abundance, Diversity and Taxonomic Composition

Total abundance, diversity and taxonomic composition of birds, reptiles and ants were analysed separately, each as a function of the experimental model above. Abundance per trap lines (for reptiles and ants) were pooled per site, excluding reptile recaptures. Bird abundance was mean abundance per site averaged across the morning and afternoon surveys. Ant abundance and ant diversity used micro‐pit data only, whilst composition used ant captures from micro‐pits and reptile pitfalls combined. Ant abundance in each vial was capped at 100 individuals per genus to reduce the influence of trap placement, in case this was near a nest or foraging trail. Taxa diversity was calculated using Shannon's effective number of species (Jost 2018, exp.(H′) in *vegan* Oksanen et al. 2020). Bird and reptile abundance and diversity were tested using linear mixed models (*lme4*, Bates et al. 2015). When modelling ant abundance and diversity, *Site Pair* (random effect) had near zero variance and was contributing to a singular fit; thus, we removed *Site Pair* from the model, which therefore resulted in a generalised linear model (negative binomial family, log link). Multivariate bird and reptile species composition was square‐root transformed, whilst ant genera composition was fourth‐root transformed to further down weight species with high counts and reflect more of the community composition incorporating rarer taxa. Each taxon was then analysed using PERMANOVA and PERMDISP to test for differences in taxonomic composition or homogenisation, and if differences were detected, post hoc SIMPER analysis in PRIMER‐e identified which species had the highest percent contribution to the average community dissimilarity with invasion and whether their average abundance was higher in invaded or native sites.

#### 2.5.2 Detecting Impacts of Invasion Using a Functional Approach

To assess the impact of invasion on faunal communities at the functional level we tested for differences in composition (PERMANOVA) and homogenisation (PERMDISP) of the fauna communities based on aggregated functional groups. For birds and reptiles, aggregated abundances were square‐root transformed, and three resemblance matrices were analysed separately for each taxon: birds (1) diet × movement, (2) diet × foraging strata and (3) foraging strata × movement; and reptiles (4) diet × body size, (5) diet × habitat use and (7) habitat use × body size. For ants, ant functional group abundances were fourth‐root transformed. Ordinations were plotted using Principal Coordinates Ordination (PCO) in PERMANOVA add‐on to assist with interpretation.

If significant differences in community composition delineated by aggregated functional groups were detected with invasion, post hoc SIMPER analyses identified their contribution to community dissimilarity. To assess whether these contributions aligned with predictions in the conceptual model, including whether any axes were moderating the outcome of the other axes for two‐way groups, the SIMPER one‐way outputs (Appendix A: Tables A6, A7, A10, A12) were visualised as two‐way tables along the axes of diet, habitat use and scale for each taxon (Figures 7, 9, 10). Each cell was shaded to indicate strongest vs. weakest differences (largest to smallest percent contribution to average dissimilarity, dark to light shading) and coloured according to higher average abundance in native (blue) or invaded sites (red) in the SIMPER analysis. Colours do not indicate statistical differences in abundance of each functional group but indicate trends toward groups more associated with invaded or native sites. Many groups occurred at few sites or in low abundance due to the dry climatic conditions, which precluded additional statistical testing of individual group abundance (e.g., linear models).

#### 2.5.3 Identifying Habitat Differences (Resource Changes) Associated With Invasion

To identify the key habitat and resource variables that differed with invasion at our field sites to assist with interpretation of fauna trends, we assessed plant community composition and five habitat variables as a function of the experimental model as above. Resultant plant community composition and dispersion (buffel grass + understorey + overstorey plant species) was analysed using PERMANOVA and PERMDISP, using the mid‐point of each cover class as the abundance value, and square root transformed. Percent cover of buffel grass, litter, bare ground, native understorey plants and overstorey plants was modelled using generalised linear mixed models (proportion format, beta regression, logit link, *glmmTMB* Brooks et al. 2017). A very small constant (0.000001) was added to the data to conform to parameters of beta regression (no absolute zeros). Coarse woody debris was < 1% cover and strongly correlated with overstorey woody cover and was not analysed.

## 3 Results

### 3.1 Habitat Differences Between the Invaded and Native Sites

The habitat of invaded and native sites was differentiated by total cover of native understorey plants, total cover of buffel grass and plant composition. Invaded and native sites were selected based on a priori defined thresholds of buffel grass cover, and the field survey confirmed buffel grass cover was on average eight times higher in invaded sites (mean cover 36% ± 8% s.e.) compared to native sites (5% ± 3% cover, values reported for all taxa sites, *p* < 0.001 all taxa sites and bird sites, Appendix A: Table A1). Native understorey cover averaged 30% ± 4% at native sites but was four‐fold less at invaded sites, with an average of 6% ± 3% cover (*p* < 0.001). Plant community composition differed between invaded and native sites (PERMANOVA invasion status *p* ≤ 0.007) and was more homogenised at invaded sites in the west region (PERMDISP invasion × region *p* ≤ 0.018, Appendix A: Table A2). Bare ground cover (~44%) and litter cover (~9%) showed no difference between invaded and native sites on average (*p* ≥ 0.32, Appendix A: Table A1). Overstorey vegetation was one of the criteria we used to select sites within a pair and, as intended, overstorey plant cover did not differ significantly between invasion status (~8% cover, *p* > 0.60). Regions showed minimal differences in habitat structure apart from litter and overstorey cover for the ‘all taxa’ sites (Appendix A: Table A1).

### 3.2 Bird Community Responses to Invasion

**FIGURE 4:**
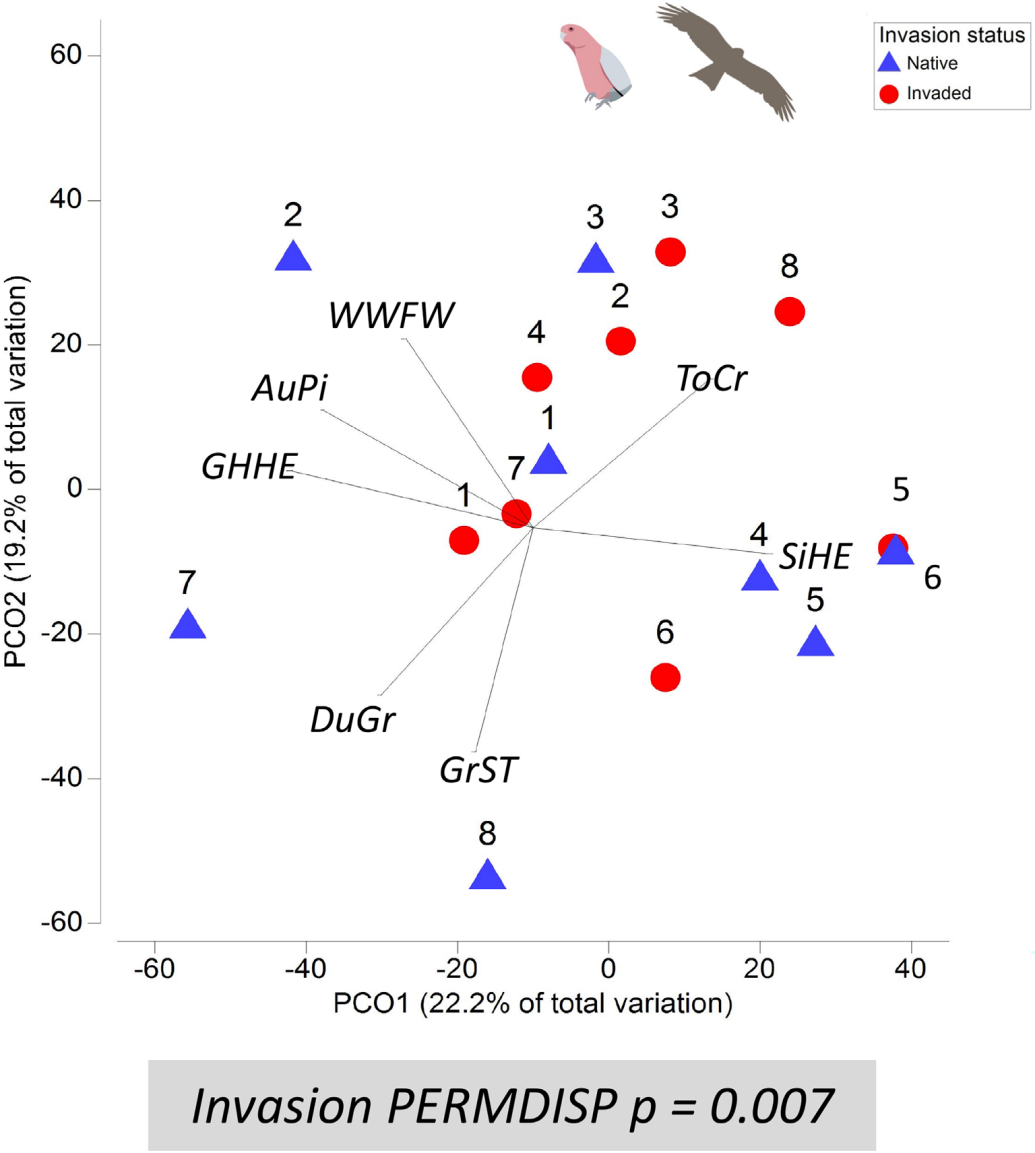
Principal components ordination (PCO) of bird species composition at native sites (blue triangles) and buffel grass invaded sites (red circles) with paired sites (same plant community) indicated by matching numerals. PERMDISP analysis found significantly smaller dispersion for invaded sites. Vectors show bird species with correlation > 0.65. Sites 7 and 8 were rocky hills. Species matrix is mean bird abundance (AM + PM surveys), square root transformed, Bray–Curtis similarity, (*n* = 16). AuPi = Australasian Pipit, DuGr = Dusky Grasswren, GHHE = Grey‐headed Honeyeater, GrST = Grey Shrike‐thrush, ToCr = Torresian Crow, SiHE = Singing Honeyeater, WWFW = White‐winged Fairy‐wren.

**FIGURE 5:**
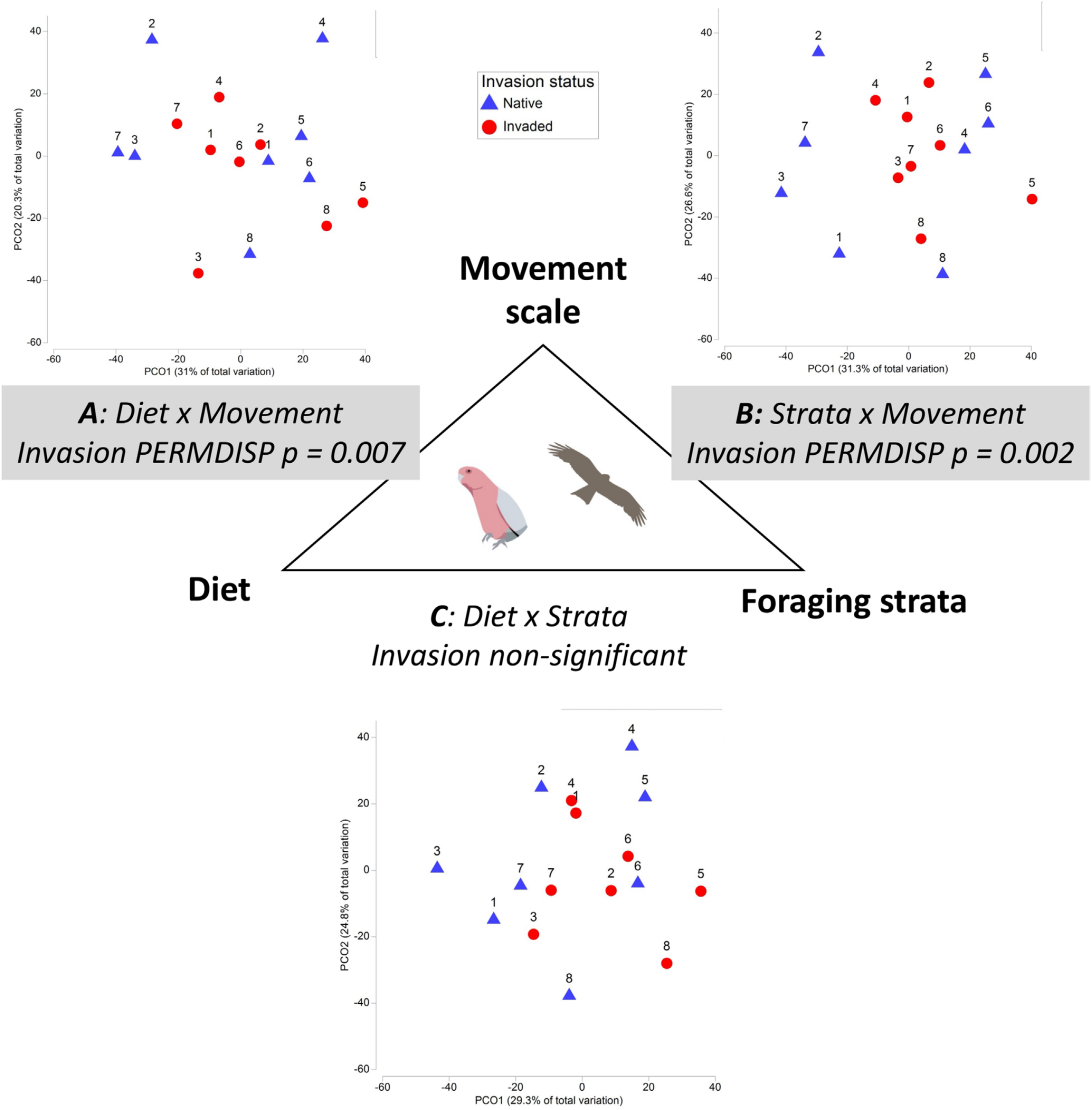
Differences in the functional compositional of bird communities in native (blue triangles) and paired invaded sites (red circles) with site pairs indicated by matching numerals. Significant dispersion differences were detected for birds grouped by (A) diet × movement, and (B) foraging strata × movement, but not (C) diet × foraging strata. Principal components ordinations (PCO) are based on bird abundance aggregated into two‐way functional groups, square root transformed, with a Bray‐Curtis percentage difference resemblance matrix, (*n* = 16).

In total 39 species and 324 individual birds were observed across 16 sites. Bird assemblages at native and invaded sites differed taxonomically and in functional group structure. Across both regions, bird taxonomic composition was more homogenised (smaller dispersion) at invaded sites compared to native sites (PERMDISP *p* = 0.005, Figure 4, Appendix A: Table A4), despite the site pairs representing broadly different plant communities. Bird functional group structure was more homogenised at invaded sites, as indicated by reduced multivariate dispersion when the bird community was delineated by diet × movement traits (PERMDISP *p* = 0.005) and delineated by foraging strata × movement traits (PERMDISP *p* = 0.002, Figure 5, Appendix A: Table A4). Neither total abundance nor species diversity of birds differed significantly with invasion in either region (Appendix A: Table A13).

**FIGURE 6:**
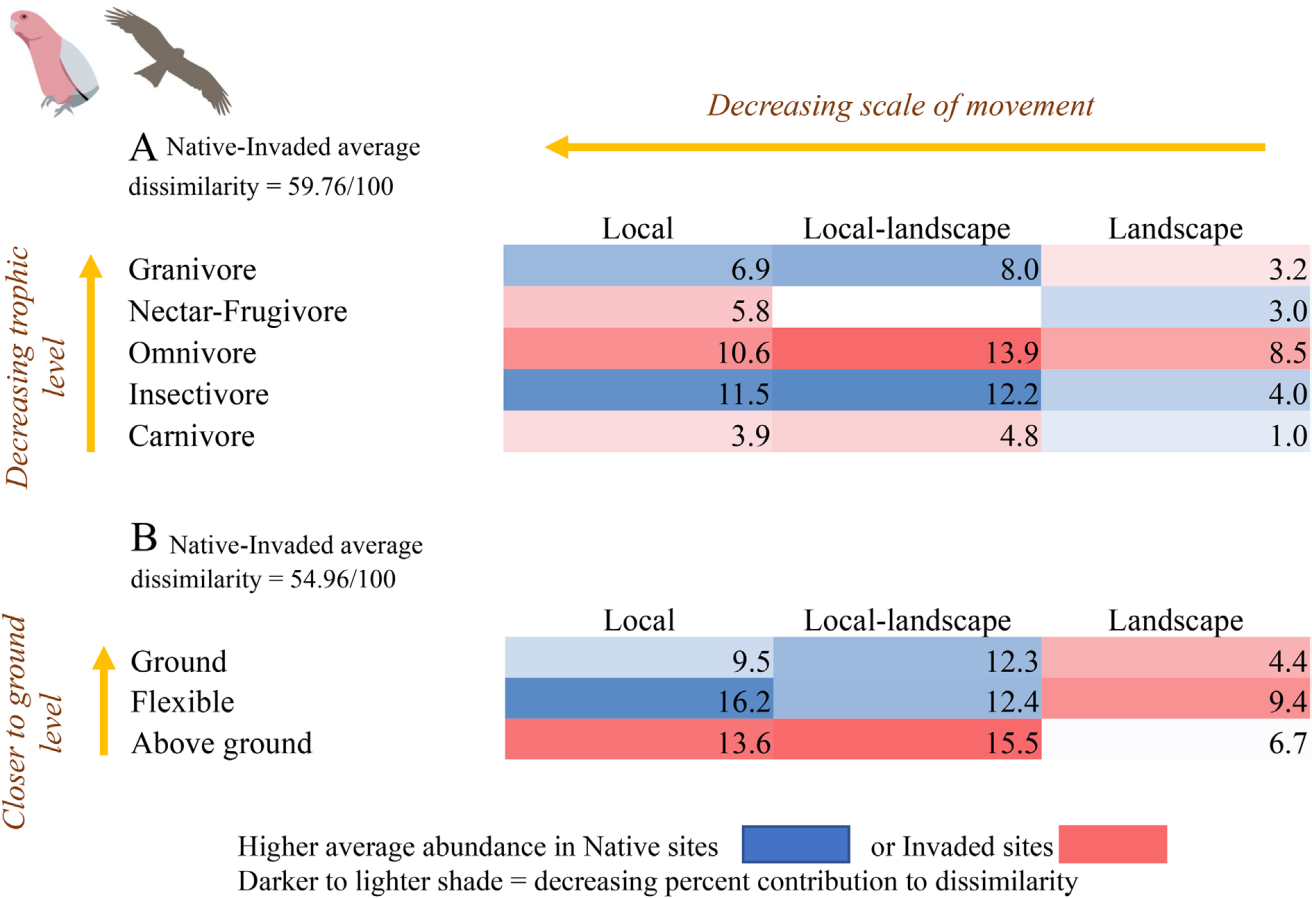
Percent contribution of bird two‐way functional groups to average community dissimilarity between invaded and native sites (PERMDISP < 0.007), based on post hoc SIMPER analysis. Groups are tabulated in shaded matrices ordered by trophic level, foraging level and spatial scale. Groups more characteristic of native sites (blue) contrast with those characteristic of invaded sites (red), with darker shading indicating a larger contribution to community dissimilarity.

Bird species characteristic of native sites included insectivorous species of varying mobility, the Dusky Grasswren (local scale), Australian Pipit (local‐landscape) and Rufous Whistler (landscape) and granivorous Zebra Finch (local‐landscape) (SIMPER Appendix A: Table A5). Bird species defining the more simplified invaded bird community included omnivorous birds with above ground or flexible foraging including Torresian Crow, Black‐faced Cuckooshrike, Yellow‐throated Miner and Singing Honeyeater (SIMPER Appendix A: Table A5). In the analysis by functional groups, bird communities at native sites were characterised by granivores that moved at local to local‐landscape scales, and insectivores regardless of mobility, in contrast to invaded sites where omnivores dominated (Figure 6). Native sites were characterised by birds that used the ground wholly or partly for foraging (ground or flexible) that operated at local to local‐landscape scales, whilst arboreal foraging groups persisted at invaded sites (Figure 6). These interpretations are based on which groups contributed most to community dissimilarity (percent contributions, as reported in Figures) and whether they were more abundant in native or invaded sites (SIMPER Appendix A: Tables A6 and A7, see methods). Birds active at the landscape scale contributed the least to differences between invaded and native sites for all diet or foraging groups (lighter shading, Figure 6). Carnivores and nectar‐frugivores were not strong contributors at any scale.

### 3.3 Reptile Community Responses to Invasion

There were 30 reptile species sampled across 12 sites, with 128 individual captures. Our sampling coincided with the driest season on record (Bureau of Meterology 2021, Ryan‐Colton et al. 2024) and, as a result, abundance and diversity of reptiles were underrepresented in our samples. Only four species (*Lerista labialis, Ctenophorus isolepis, Ctenotus leonhardii, Tympanocryptis centralis*) had five or more captures and 14 species were captured only once (Appendix A: Table A8).

**FIGURE 7:**
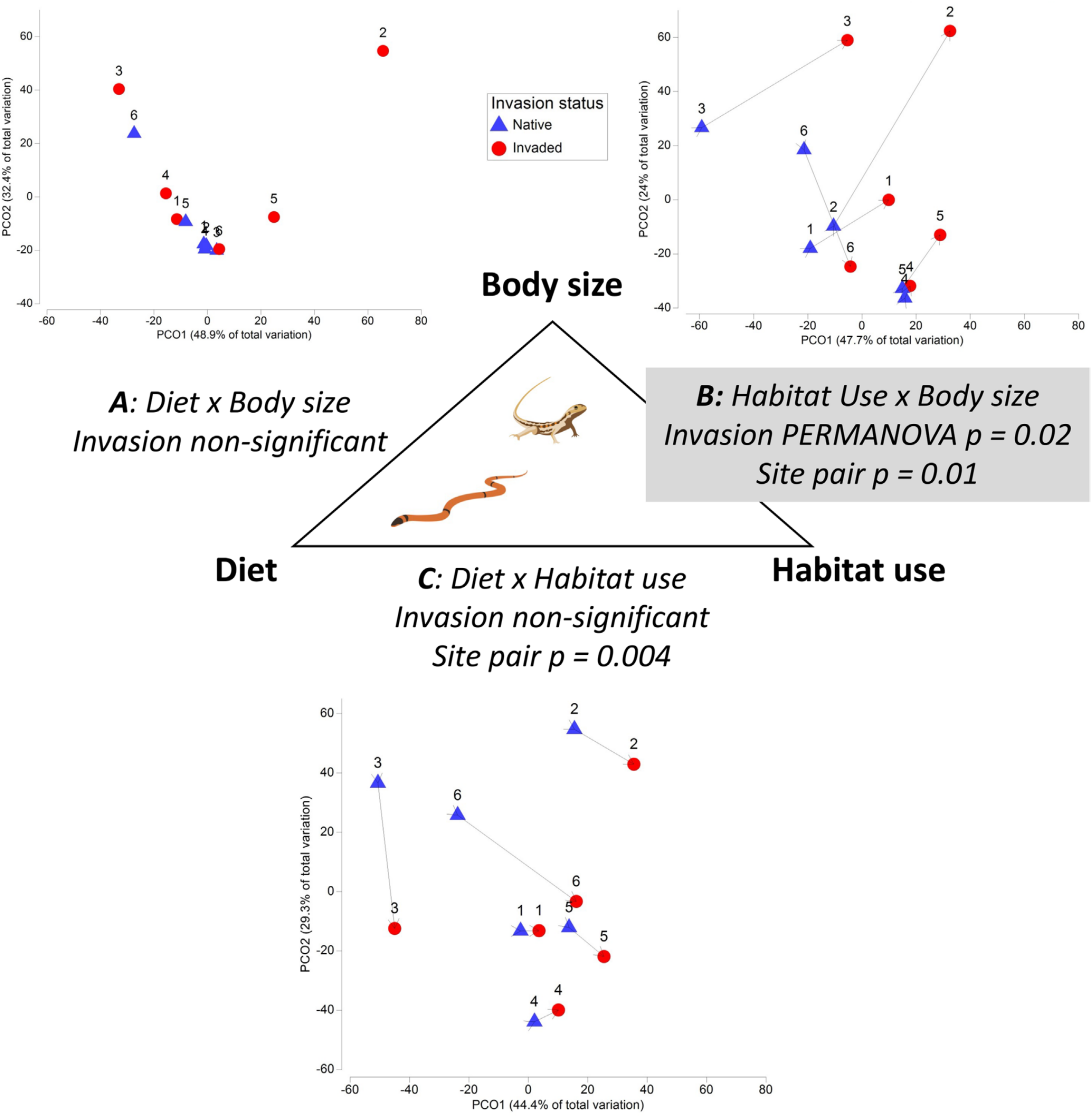
Differences in the functional compositional of reptile communities in native (blue triangles) and paired invaded sites (red circles) with site pairs indicated by matching numerals Significant differences in functional composition were detected for reptiles grouped by (B) habitat use dependent on body size, but not (A) diet dependent on body size or (C) diet depending on habitat use. Trajectory lines indicate a significant Site Pair factor in the model (B, C). Principal components ordination (PCO) plots are based on reptile abundance aggregated into two‐way functional groups, square root transformed, with a Bray‐Curtis percentage difference resemblance matrix, (*n* = 12).

Reptile communities differed significantly in their functional group structure at invaded compared to native sites, when grouped by reptile body size × habitat use (PERMANOVA *invasion status p* = 0.02, Figure 7) but not for two‐way groups involving diet (PERMANOVA *invasion status p* > 0.05 Appendix A: Table A9). Observed reptile taxonomic composition varied considerably between site pairs (*Site pair p* = 0.001, 37% ECV, Appendix A: Table A9), but there was no clear difference in species composition with invasion (*p* > 0.24). For reptiles, neither total abundance nor recorded species diversity differed with invasion in either region (Appendix A: Table A13). However, when aggregating reptiles into two‐way functional groups, invaded and native sites differed significantly in the functional composition of reptiles, depending on reptile body size × habitat use (PERMANOVA *invasion status p* = 0.02, Figure 7) but not for two‐way groups involving diet (PERMANOVA *invasion status p* > 0.05 Appendix A: Table A9).

**FIGURE 8:**
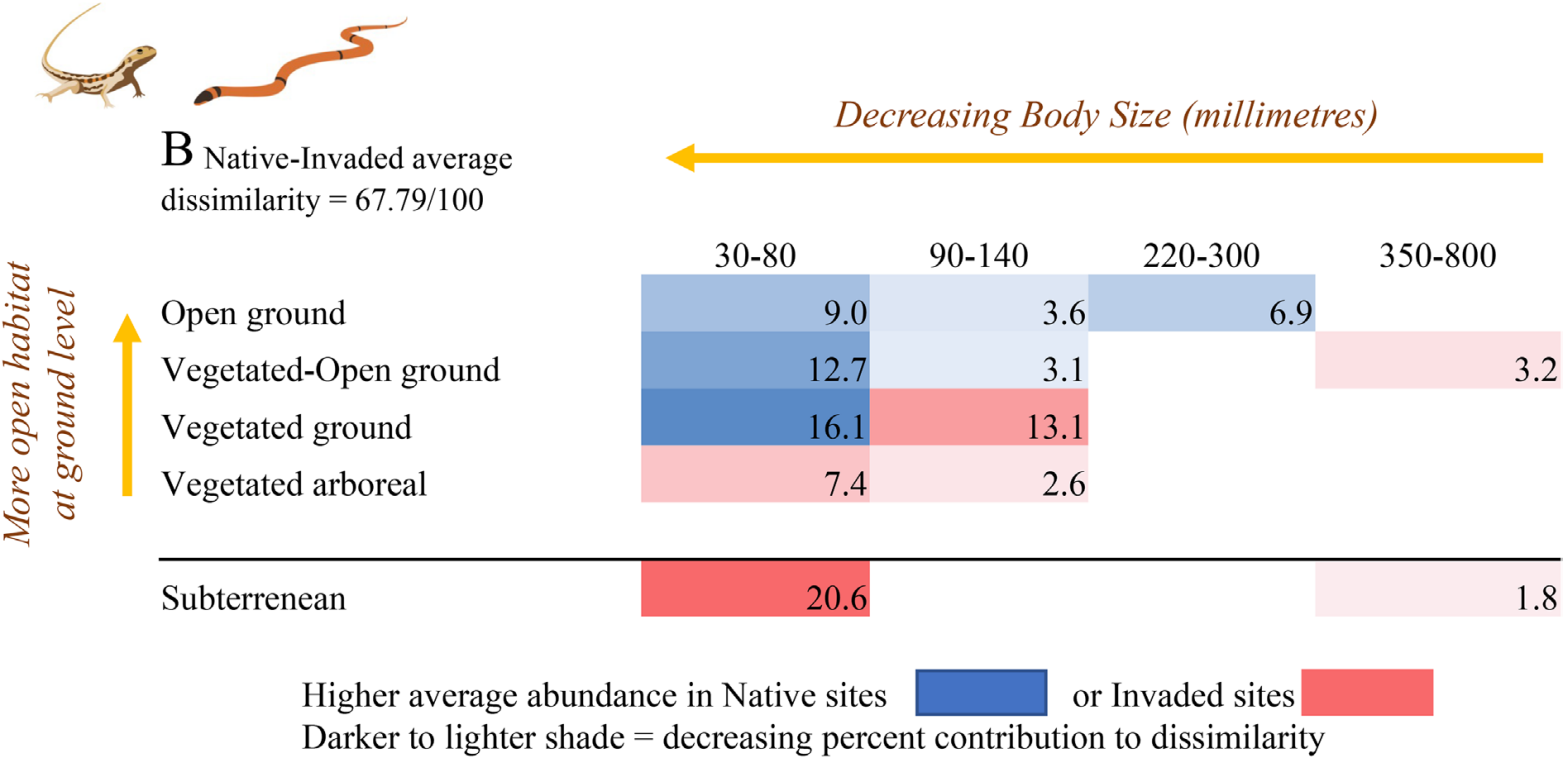
Percent contribution of reptile two‐way functional groups to average community dissimilarity between invaded and native sites (PERMANOVA *p* = 0.02), based on post hoc SIMPER analysis. Groups are tabulated in a shaded matrix ordered by habitat openness combined with proximity to ground level, and body size. Subterranean reptiles, placed below the line, were excluded from the habitat continuum because their interaction with terrestrial habitats was unclear. Groups more characteristic of native sites (blue) contrast with those characteristic of invaded sites (red), with darker shading indicating a larger contribution to community dissimilarity.

In line with predictions, reptiles with the smallest body size (30–80 mm) contributed most to the community differences between invaded and native sites (darker shading, Figure 8, Appendix A: Table A10). Small terrestrial (ground‐level) reptiles showed more association with native sites regardless of their open or vegetated habitat preference (blue shading, Figure 8). These groups included three species of ground geckos (e.g., *Rhynchoedura ornata* ), two terrestrial dragons (e.g., *Tympanocryptis centralis* ) and seven skinks (e.g., *Ctenotus* spp. *Carlia triacantha, Menetia greyii*) (Appendix A: Table A8). However, small reptiles that used arboreal (e.g., *Gehyra* spp.) or subterranean habitats (*Lerista labialis*), persisted in invaded sites (red shading, Figure 8). Reptiles of moderate body size (90–140 mm) persisted in invaded sites if they preferred vegetated habitats (e.g., Vegetated ground, *Delma borea, Ctenotus pantherinus*) but not if they preferred open habitats (e.g., *Ctenophorus nuchalis, Nephrurus levis*). Even at larger body size (220‐300 mm) reptiles that use open habitats were associated with native rather than invaded sites (e.g., *Lialis burtonis, Pygopus nigriceps*) (Figure 8).

### 3.4 Ant Community Response to Invasion

There were 20 ant genera in eight functional groups recorded across 12 sites, with 2755 ants sampled (2571 ants from micro‐pitfalls, 184 from reptile pitfalls). Dominant Dolichoderinae *Iridomyrmex* was most abundant (48.3% of captures), followed by Hot Climate Specialists *Melophorus and Monomorium* (~20% each), General Myrmicinae *Crematogatser* and *Pheidole* (~3% each), Opportunist *Rhytidoponera* and Subordinate Camponoti *Calomyrmex* (~1% each) and the remaining genera were represented by less than 1% of captures.

**FIGURE 9:**
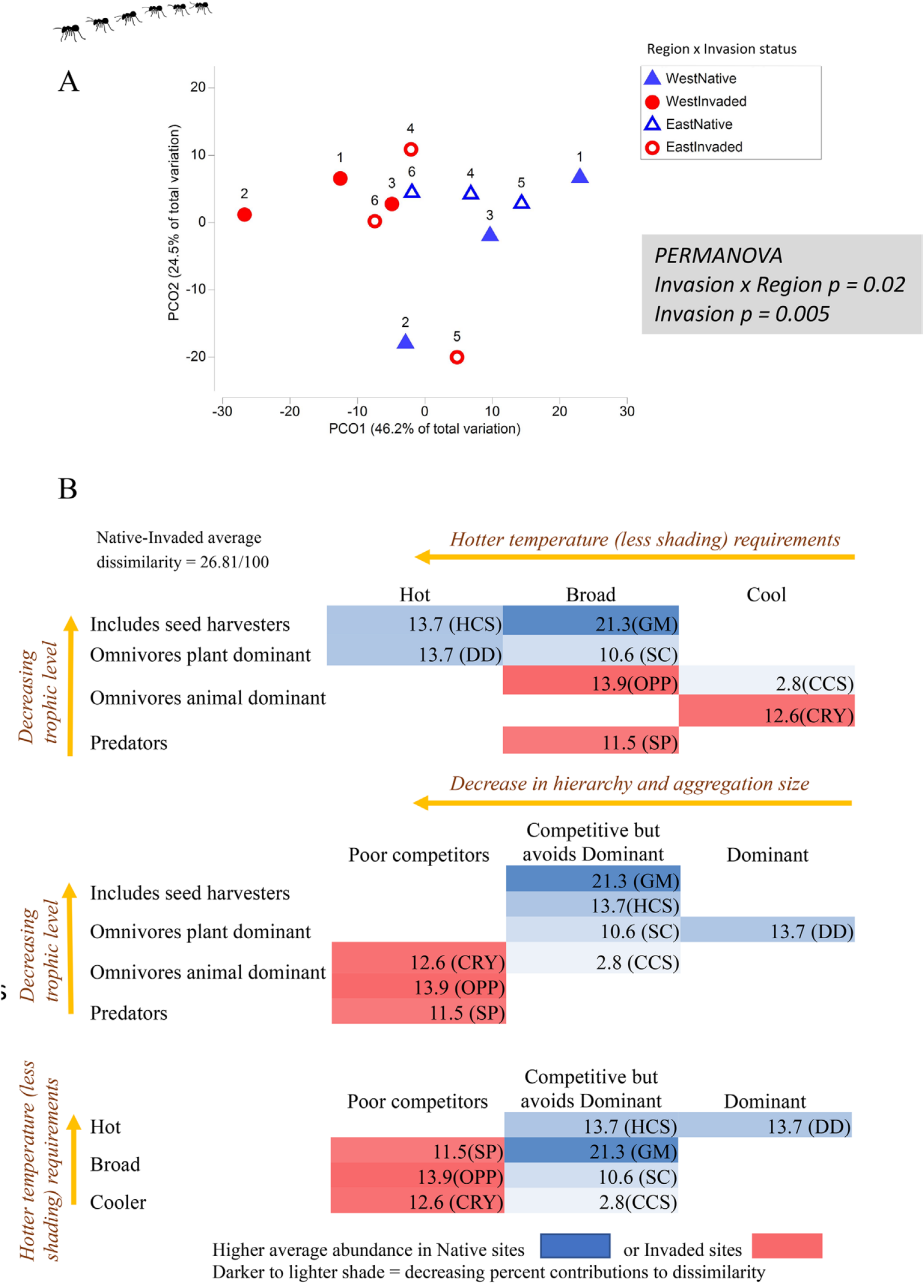
(A) Ant community composition aggregated by functional groups, for invaded (red circles) and matched native sites (blue triangles), with site pairs labelled numerically. Ants differed significantly by invasion status (red vs. blue), and interaction of region and invasion (shapes, as per legend, PERMANOVA *p* ≤ 0.02). Principal components ordination (PCO) plot is based on ant functional group abundance, fourth root transformed, with a Bray‐Curtis percentage difference resemblance matrix, (*n* = 12). (B) Percent contribution of ant functional groups to average community dissimilarity between invaded and native sites (SIMPER analysis). Data are tabulated in three shaded matrices to visualise trends associated with trophic level, temperature requirements and aggregation size in the ant hierarchy. Groups more characteristic of native sites (blue) contrast with those characteristic of invaded sites (red), with darker shading indicating a larger contribution to community dissimilarity. Group name abbreviations are found in the methods section.

Total ant abundance was over 50% less on average in invaded compared to native sites (micro‐pitfall traps only, exp. (estimate) = 0.41 [0.25–0.68, 95% confidence interval]). Ant functional groups also differed significantly between invaded and native sites (all trapping methods, PERMANOVA *invasion status p* = 0.005, Figure 9), and the difference was more distinct in the West region (PERMANOVA *invasion status* × *region*, *p* = 0.02, Figure 9, Appendix A: Table A11). Most variation in ant taxonomic composition related to site characteristics (most variability between site pairs, highest ECV 14%) and no taxonomic differences were detected with invasion status (Appendix A: Table A11). Ant diversity did not differ with invasion in either region (Appendix A: Table A13). However, community differences were apparent when ant genera were aggregated into functional groups (all trapping methods, PERMANOVA *invasion status p* = 0.005, Figure 9), and the difference was more distinct in the West region (PERMANOVA *invasion status* × *region*, *p* = 0.02, Figure 9, Appendix A: Table A11).

General Myrmicinae ants (seed harvesting *Pheidole* and *Crematogatser*) contributed most to community dissimilarity (~21%) and characterised native sites (blue shading, Figure 9, Appendix A: Table A12). Other groups associated with native sites were groups that are active at hotter temperatures: Dominant Dolichoderinae (*Iridomyrmex*) and Hot Climate Specialists (~14% contribution each). Hot Climate Specialists included partial or complete seed harvesters (e.g., *Melophorus* and *Meranoplus* respectively), and along with Subdominant Camponoti (~11% contribution, e.g., *Camponotus*), all groups that characterised native sites were of lower trophic level (Figure 9). Functional groups that were higher in the ant hierarchy (larger aggregation sizes, dominant competitors) were more associated with native sites (blue shading, Figure 9). In contrast, invaded sites were characterised by Opportunists (e.g., *Rhytidoponera, Tapinoma*), Cryptic species (e.g., *Pseudoneoponera*) and Specialist Predators (e.g., *Odontomachus*) which are more solitary ants, of higher trophic levels but poorer competitors compared with ants that aggregate (red shading, Figure 9).

## 4 Discussion

**FIGURE 10:**
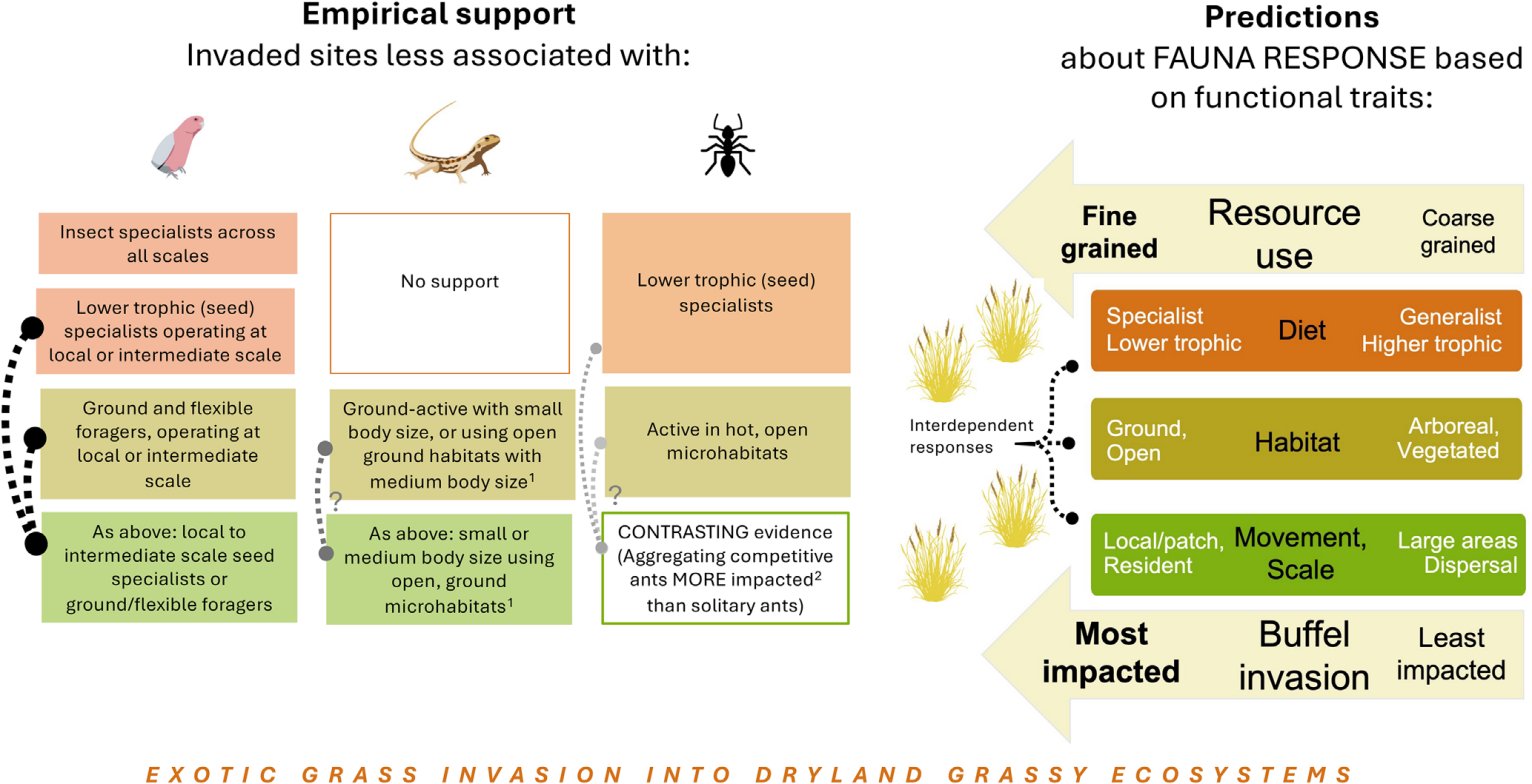
Empirical support across three major faunal groups—birds, reptiles and ants—for the predictions in our functional framework, about which groups of fauna are most impacted by exotic grass invasion into dryland ecosystems (trait‐filtering). The analysis considered fauna response based on combinations of traits (interdependence), which was important for fauna communities to varying degrees (dashed lines, black to grey, high to low confidence). ^1^Aboreal and large bodied reptiles under‐sampled, more testing recommended. ^2^Impacts on aggregating competitive ants (in contrast to predictions) are likely due to these groups having strong habitat preferences for hot open microhabitats and/or being lower trophic, including seed specialist ants.

Our findings support the hypothesis that buffel grass invasion alters the functional structure of faunal communities and highlight the value of trait‐based approaches for generalising invasion impacts. Bird, ant and reptile communities all showed functional differences with invasion, whereas only isolated differences in total abundance or taxonomic composition were detected for any taxon, and no effects on species diversity were observed (Table 1) supporting our second hypothesis that functional approaches more readily detect community change. Invaded sites were characterised by fewer fauna that use open ground‐level habitats, and fewer fauna with specialised diets (Figure 10), with partial support for our prediction that responses would be moderated by the spatial scale at which species access resources. Detecting these multi‐taxa trait‐based patterns across diverse vegetation types, two regions and very dry survey periods, when activity and abundance were low, suggests that buffel grass invasion has substantial and generalisable impacts on fauna in central Australia, with likely relevance to other exotic grass invasions in comparable dryland ecosystems.

**TABLE 1:** Comparison of different community metrics used to detect statistical differences in three major fauna communities between native sites and sites invaded by buffel grass in dryland grassy ecosystems.

Community metric	Birds	Reptiles	Ants
Functional group composition	Homogenisation PERMDISP *p* = 0.002	Compositional shift PERMANOVA *p* = 0.02	Compositional shift PERMANOVA *p* = 0.005
Taxonomic composition	Homogenisation PERMDISP *p* = 0.005	ns	ns
Total abundance	ns	ns	Reduced by 50% (GLMM)
Diversity^a^	ns	ns	ns

Abbreviations: GLMM, generalised linear mixed model; ns, non‐significant; PERMANOVA, permutational multivariate analysis of variance; PERMDISP, permutational analysis of homogeneity of multivariate dispersion.

^a^ Shannon effective number of species, exp.(H′).

### 4.1 Functional Approach Versus Diversity, Abundance or Taxonomic Metrics

The more consistent detection of invasion impacts using functional metrics (*p* ≤ 0.02; Table 1) adds to mounting evidence that species richness or diversity is a less sensitive indicator of fauna response to environmental change (French and Zubovic 1997; Jones et al. 2017; Caddy‐Retalic et al. 2018). Detecting taxonomic signals of community change can be difficult in heterogeneous arid landscapes, where ants, birds and reptiles often have strong associations with distinct plant communities (Pavey and Nano 2009; Morton et al. 2011; Caddy‐Retalic et al. 2018). This high between‐community turnover means that different habitat types support distinct species assemblages, reducing the likelihood of detecting consistent taxonomic responses across habitats. Species with similar functional roles, however, are often represented across communities. For instance, Cody (1994) identified a core group of birds that characterise Australian *Acacia* shrublands, noting that some species are replaced by ecologically similar species in different regions. Consideration of functional groups rather than individual species therefore reduces this variation, making it easier to identify compositional shifts in fauna communities that are of ecological significance (Tischler et al. 2013).

In the arid zone, Read et al. (2015) likewise observed divergent trends in the abundance of distinct guilds of arid birds in response to environmental disturbance, whilst richness remain unchanged, which is consistent with the variable taxonomic responses we detected. We did detect some community differences using total abundance (ants reduced by 50%, est. = 0.41 [0.25–0.68, 95% confidence interval]) and taxonomic metrics (bird community homogenisation, *p* = 0.002). We suggest these impacts were more readily detected for birds and ants due to the large sample size for these groups compared to reptiles. Birds had more site replicates and were sampled in both plains and rocky hills, whereas reptiles and ants were sampled only in plains. On invaded hills, buffel grass had largely replaced native hummock grasses (*Triodia* spp.), and fewer *Triodia*‐specialist birds (Dusky Grasswren) were present. Other specialist species (the insectivorous Australasian Pipit and granivorous Zebra Finch) occurred on both plains and hills, and were consistently lower in abundance at invaded sites across both habitats. The inclusion of hill sites therefore strengthened the taxonomic signal for birds both by increasing number of sampled sites and because invaded hills showed particularly strong species‐level responses. For ants, sample abundances in micropits were relatively high compared with observations of birds and reptiles, especially during the extremely dry conditions of our survey. With these sample sizes, the 50% reduction in ant abundance was easier to detect in line with similar reductions that have been reported in dryland ecosystems (Kutt and Fisher 2010; Bonney et al. 2016). Our finding that bird assemblages were more taxonomically (and functionally) homogeneous at invaded sites (*p* ≤ 0.005) adds to evidence that plant invasions are contributing to biotic homogenisation globally (Olden and Poff 2003; Clavel et al. 2011).

### 4.2 Fauna Response According to Species Function

#### 4.2.1 Multi‐Taxa Impacts: Ground‐Active Fauna That Use Open Habitats, Especially Operating at Smaller Scales

Invasion was associated with consistent trait‐filtering for fauna that use open, ground‐level habitats, which was moderated to some degree by bird movement patterns and potentially reptile body size (Figure 10). Ants that use open (hot) microhabitats (28% contribution to ant community dissimilarity), small ground‐dwelling reptiles (38% contribution to reptile community dissimilarity) and birds that operate at local to local‐landscape scales and forage wholly or partially on the ground (50% contribution to bird community dissimilarity) characterised native rather than invaded sites (Figure 10), consistent with negative impacts on terrestrial (ground‐active) fauna due to biological invasions globally (Marino et al. 2022).

Birds that are flexible or ground foragers have been found to spend less time on the ground or foraging in areas dominated by exotic grasses (Sands et al. 2011; Young and Schlesinger 2014; Fulbright et al. 2019). Reduced foraging time may be due to altered food availability in the invaded understorey (e.g., seeds; Wright et al. 2020, Ryan‐Colton 2025) or altered habitat structure that affects foraging cues or physical movement (Steidl et al. 2013). Importantly, in our study, birds that made even transient use of habitat (either through dispersal or temporal variability, that is, our definition of local‐landscape birds) contributed along with local birds to community differences, suggesting that impacts of buffel grass invasion at the patch scale are not restricted to resident birds alone. Although fauna mobility is well recognised in theoretical frameworks especially regarding foraging behaviour (e.g., Ritchie 1998), it has received relatively little attention in relation to biological invasions (but see Martin and Murray 2011). Movement patterns appear to be part of the trait‐filtering underway due to exotic grass invasion, at least for arid zone birds, potentially because this group uses resources at vastly different scales (Tischler et al. 2013; Gibson et al. 2022).

Ants are intricately coupled to structurally determined thermal regimes (Roeder et al. 2022), with some groups reliant on open habitat structure that allows for hotter soil temperatures. That Hot Climate Specialists (e.g., *Melophorus* spp.) were not favoured by buffel grass invasion in our study (14% contribution to ant community dissimilarity) aligns with previous studies (Smyth et al. 2009; Bonney et al. 2016), and the sensitivity of these temperature specialists to habitat change (Read and Andersen 2000; Santos et al. 2021). However, our study shows buffel grass invasion also disadvantaged Dominant Dolichoderinae (i.e., *Iridomyrmex* spp., 14% contribution), the most ecologically dominant and abundant ant group in arid Australia (Andersen and Patel 1994), which contrasts with Bonney et al. (2016), who found no response from Dominant Dolichoderinae. This disparity could be attributed to the different stages of invasion and designs of these studies: Bonney et al. (2016) compared invaded sites to sites where buffel grass was removed and native vegetation had been restored. It is possible that population bottlenecks of certain fauna (including *Iridomyrmex* spp.) occurred during the initial invasion, from which populations had not recovered when native vegetation was restored (see discussion in Schlesinger et al. 2020). The interpretation of results from our study, where native sites had never been invaded, is more straightforward and suggests *Iridomyrmex* spp., along with Hot Climate Specialists, are significantly reduced at invaded sites.

Reptile trends should be interpreted with caution due to the low capture rates and low representation of some components of the reptile community. Arboreal species and larger‐bodied reptiles were underrepresented due to our pitfall trapping method, which is strongly biassed toward capturing small ground‐active species. Our results showed terrestrial (ground‐active) reptiles were most impacted by grass invasion into the understorey (38% contribution to community dissimilarity), in line with our predictions, and this response is likely due both to preferential sampling and to true ecological impact. Earlier research has found most reptile species sampled in pitfall traps had lower abundance in buffel‐invaded areas (Schlesinger et al. 2020), and other ground‐layer disturbances (i.e., grazing) also have been found to negatively impact terrestrial rather than arboreal reptiles (Kutt and Fisher 2011; Neilly et al. 2017). In our study arboreal (10% contribution, e.g., *Gehyra* spp.) and subterranean species (22% contribution, e.g., *Lerista labialis*, *Anilios endoterus* ) characterised invaded sites, which was expected given our experimental design, where we deliberately chose paired sites with contrasting understorey (native versus invaded), but similar overstorey cover (*p* > 0.60) and presumably similar (although untested) subterranean conditions. However, for arboreal reptiles this association with invaded sites is based on a relatively small contribution to community dissimilarity (10%), which may reflect insufficient sampling and thus lack of statistical power. Further testing of arboreal groups is recommended in modified sampling frameworks and experiments that include overstorey change (see discussion below). Reptile body size was potentially one moderator of impact, which also needs further investigation due to the underrepresentation of reptiles larger than 220 mm (10% contribution). Larger body size may enable fauna to seek optimal thermal regimes over a wider area, as invasive plant monocultures can diminish structural and thermal heterogeneity (Valentine et al. 2007; Garcia and Clusella‐Trullas 2019; Lara‐Resendiz et al. 2022), which could significantly disadvantage smaller ectothermic species that are less able to move away from suboptimal thermal regimes.

Bare ground cover did not vary on average between invaded and native sites in our study, and yet there was a consistent response across taxa from ground‐active (birds, reptiles) and open microhabitat specialists (reptiles, ants). Buffel grass replaced native understory vegetation (*p* < 0.001) and altered and homogenised plant composition (*p* ≤ 0.007), but bare areas remained comparable within paired‐plots on average. Therefore, ground and open habitat specialists may be responding to more complex structural alterations and/or shifts in food resource availability, stemming from changes to vegetation heterogeneity (Valentine et al. 2007; Litt and Steidl 2010). Even with comparable bare ground cover, other biophysical changes associated with bare ground may include (a) the temperature, nutrient or moisture profiles in bare inter‐tussock spaces (e.g., Leite et al. 2022) or (b) altered connectivity of bare areas. For example, buffel grass tussocks are on average taller and denser than native tussock grasses (Marshall et al. 2012), potentially creating more shading at ground level in inter‐tussock spaces, and cooler temperature profiles inside tussocks. Buffel grass becomes particularly dense when invading fertile alluvial soil, following regrowth after fire, and when longer periods have elapsed since invasion (Clarke et al. 2005; Schlesinger et al. 2013, 2020). In such areas, further impacts on ground‐dwelling fauna could be expected.

#### 4.2.2 Multi‐Taxa Impacts: Fauna With Specialist Diets

Impacts on diet‐specialists included specialised consumers of seed across taxa—birds and ants—and insectivorous birds regardless of their mobility. Impacts on seed consumers are consistent with impacts of plant invasion on plant‐specialists in other ecosystems (French and Zubovic 1997, French and Major 2001). For ants, negative impacts on predominantly granivorous groups, including General Myrmicinae (*Pheidole*) and Hot Climate Specialists (*Monomorium, Meranoplus* and partial seed harvesters *Melophorus*), have been reported when buffel grass invades similar arid ecosystems Bonney et al. (2016). Ant communities are known to be structured by their habitat structural requirements (A. Andersen 1995, 2019), but our finding that seed specialist ants (the largest contribution to ant community dissimilarity by any functional group; 21%) suggests that availability or access to seed resources is an additional likely mechanism by which grass invasion affects ant communities. Granivorous birds that operated over local (e.g., Galah) to local‐landscape scales (e.g., Zebra Finch) were more associated with native sites (together contributing 15% to community dissimilarity) in line with predictions. The infrequent observation of landscape granivores in our study due to the dry conditions (3% contribution, highly mobile and eruptive species such as Budgerigar), suggests their lack of ecological response may reflect limited sampling and statistical power for this group, rather than a true absence of impact. Overall, the impacts on seed consumers operating at all but landscape scales contrast with previous suggestions that plant invasions do not affect grassland trophic structure (McCary et al. 2016).

At invaded sites, the homogenised bird community contained fewer specialist insectivores (28% contribution to dissimilarity), whilst generalist omnivores persisted (33% contribution), regardless of the scale at which those bird guilds operated. Globally, generalists are replacing specialists in response to various stressors (Clavel et al. 2011), with insectivorous birds identified as highly threatened by biological invasions (Marino et al. 2022). In arid areas, insectivores tend to comprise a larger component of the bird community in dry periods compared to wetter periods (Tischler et al. 2013; Pascoe et al. 2021; Gorta et al. 2022). The higher presence of insectivores during our dry sampling period may therefore have intensified observed impacts on this group. It is plausible that invertebrate prey was reduced in invaded sites, given the 50% reduction in ant abundance, but other invertebrates were not sampled. Homogenisation of higher trophic level fauna can reflect resource homogenisation (Almeida‐Neto et al. 2011), to which insectivores seemed particularly sensitive in our study. Reptile diet, which includes specialist insectivores, did not emerge as a key functional trait differentiating invaded and native sites, however, reptile diet as an uninformative trait is consistent with findings from other invasion studies (Hacking et al. 2014; Abom et al. 2015).

#### 4.2.3 Impacts in Contrast to Predictions: Dominant and Competitive Ants

Impacts on ecologically dominant and competitive ant groups (62% contribution to community dissimilarity) contrasted with our predictions that aggregating groups would experience fewer impacts given they monopolise resources over a larger area than solitary ants. However, because many of these competitive ant groups are strongly tied to hotter temperature regimes or have specialist plant diets, impacts on these groups align with predictions based on habitat use or diet in our conceptual model. Invaded sites favoured poorly competitive, typically solitary ants (38% contribution to community dissimilarity), which may have become more prominent once the dominant groups were reduced significantly in abundance. These solitary groups (Opportunists, Cryptic species, Specialist Predators, e.g., *Rhytidoponera, Pseudoneoponera, Odontomachus* respectively) are also associated with dense vegetation and leaf litter, or have broader habitat preferences (A. Andersen 1995), which may support their persistence in invaded sites.

### 4.3 Application of the Functional Framework

Identifying trait‐filtering in response to defined environmental changes has broad application for community ecology and conservation, including understanding sensitivity to resource homogenisation, likely mechanisms of impact and risk assessment. The key is defining context‐dependent resource changes (Litt and Pearson 2022) to inform predictions about the multi‐faceted response of fauna, which can then be refined for different recipient ecosystems (i.e., exotic trees into forests), ecosystem processes (i.e., fire or hydrological regimes) or alternative ecosystem states through time. For example, the differences in fauna communities reported here reflect responses where only the understorey differed with invasion. We would expect further impacts on other fauna groups when overstorey shrubs and trees, which provide critical resources for nesting and foraging for a range of species, are negatively impacted over longer time‐scales (Olsson, Betancourt, McClaran, and Marsh 2012) or by fire and invasion interactions (Schlesinger and Westerhuis 2021). Scale of resource use proved a useful moderator for vertebrates. Ant hierarchy and competitive interactions are worthy of inclusion in future frameworks, rather than using ant aggregation as a measure of ‘scale’ analogous to much larger‐bodied fauna groups. Interdependent traits were important, as well as multi‐taxa evidence, which has extended other conceptual models in this field (e.g., Martin and Murray 2011; Litt and Pearson 2022). Finally, functional approaches appear sensitive enough to detect change despite considerable heterogeneity, including when fauna observations are low. This is critical, not only because low abundances are common in community ecology (particularly for vertebrates), but also because ecological impacts can be magnified when populations are at their lowest (Doody et al. 2017).

## 5 Conclusion

Buffel grass invasion is substantially altering multiple fauna communities across taxa in predictable and significant ways. Exotic grasses continue to expand across millions of hectares globally, and this fine‐grained, detailed information about the types of fauna most at risk is urgently needed to fast‐track risk assessment and management. Community‐level functional approaches appear more sensitive to detecting change across a broad range of taxa, compared to other metrics, especially in heterogeneous systems. Such trait‐based frameworks, which both identify multiple resource changes and corresponding faunal responses, can be adapted for other environmental changes in comparable systems worldwide.

## Author Contributions

**Ellen Ryan‐Colton:** conceptualization (lead), data curation (lead), formal analysis (lead), funding acquisition (lead), investigation (lead), methodology (lead), validation (lead), writing – original draft (lead), writing – review and editing (equal). **Glenda M. Wardle:** conceptualization (supporting), formal analysis (supporting), supervision (supporting), visualization (supporting), writing – original draft (supporting), writing – review and editing (supporting). **John L. Read:** conceptualization (supporting), formal analysis (supporting), supervision (supporting), writing – review and editing (supporting). **Kris French:** conceptualization (supporting), formal analysis (supporting), supervision (supporting), writing – review and editing (supporting). **Christine A. Schlesinger:** conceptualization (supporting), formal analysis (supporting), funding acquisition (supporting), investigation (supporting), supervision (lead), writing – original draft (supporting), writing – review and editing (equal).

## Funding

In‐kind and financial support was provided by APY Land Management, Central Land Council, State Herbarium of South Australia, South Australian Museum Terrestrial Invertebrates section, Department of Environment and Water, Alinytjara Wiluṟara Landscape Board, Indigenous Desert Alliance (Ten Deserts Project), Charles Darwin University, Jill Landsberg Trust and the Holsworth Wildlife Research Endowment (Ecological Society of Australia). The study was conducted with APY Executive Board approval, South Australian Scientific Permit Q26782, Northern Territory Wildlife Permit 63104 and CDU Animal Ethics Approval A18023.

## Conflicts of Interest

The authors declare no conflicts of interest.

## Data Availability

Data (Ryan‐Colton 2025) are available from Zenodo: https://doi.org/10.5281/zenodo.15803108. Aṉangu Pitjantjatjara Yankunytjatjara (APY) Organisation represents the Traditional Owners of the lands contained within the study area, and all data collected on their lands are jointly the intellectual property of Aṉangu and the authors. The APY Organisation has granted permission for future use of the dataset and publications arising from the dataset with acknowledgment of Aṉangu and the authors as Intellectual Property holders.

## Appendix A Supplementary Information, Figures and Data analysis

### A.1 Habitat Variables and Plant Community Composition

**TABLE A1:** Differences between invaded and native sites for five habitat variables. Raw data (± standard error) shown for Invasion Status and significance level shown for Invasion Status (and Region where significant) based on generalised linear mixed models (Ryan‐Colton 2025). This is the data for 12 sites (reptiles and ants, with cover assessed using point sampling of top‐down images) and 16 sites (for birds = 12 all taxa sites + 4 birds only, with cover assessed visually over one‐hectare sites).

Response variable	Data set	Raw data mean (± standard error)		Significance level for main predictors assessed by models	
		**Native Sites**	**Invaded Sites**	**Invasion Status**	Region
Buffel grass cover	12 all taxa sites	**4.6%** (± 2.9%)	**36.0%** (± 8.1%)	***p* < 0.001**	
	16 bird sites	**3.8%** (± 2.2%)	**30.2%** (± 7.1%)	***p* < 0.001**	
Native understorey cover	12 all taxa sites	**29.5%** (± 4.3%)	**5.6%** (± 2.5%)	***p* < 0.001**	
	16 bird sites	**31.4%** (± 3.5%)	**11.3%** (± 4.2%)	***p* < 0.001**	
Bare ground cover (including rock)	12 all taxa sites	46.6% (± 4.6%)	41.1% (± 3.7%)	*p* = 0.32	
	16 bird sites	35.0% (± 7.0%)	32.0% (± 6.0%)	*p* = 0.58	
Litter (percent cover)	12 all taxa sites	9.4% (± 1.9%)	9.9% (± 3.1%)	*p* = 0.32	*p* = 0.02 (higher in east)
	16 bird sites	8.7% (± 1.6%)	9.3% (± 2.3%)	*p* = 0.86	*p* = 0.006 (higher in east)
Overstorey shrub and tree cover	12 all taxa sites	8.5% (± 3.8%)	6.3% (± 3.3%)	*p* = 0.76	*p* = 0.004 (higher in east)
	16 bird sites	9.5% (± 2.9%)	7.9% (± 3.0%)	*p* = 0.60	

*Note:* Generalised linear mixed model beta regression (logit link) for percent cover of habitat variables including ~ Invasion Status (fixed) +/− Region (fixed) + Site Pair (random). Most parsimonious model +/− Region was based on model diagnostics and Delta AICc < 1. Significance level *p* was derived from likelihood ratio tests (LRT), and 95% confidence intervals. ns = non‐significant *p* ≥ 0.05.

**TABLE A2:** Multivariate PERMANOVA and PERMDISP for plant community composition at invaded and matched native sites, comprising all understorey and overstorey plant species, including buffel grass, for 12 all taxa sites and 16 bird sites (12 all taxa + 4 extra birds only).

Predictor variable	df	Pseudo‐F	P (perm)	ECV	PERM DISP *p*
*(a) all taxa sites: plant community composition*					
**Invasion Status**	1	2.45	**0.007**	18.8	**0.029**
Region	1	1.96	0.105 (MC)	23.6	0.082
**Site Pair (Region)**	4	2.38	**0.001**	31.7	
**Invasion Status × Region**	1	1.12	0.355	7.7	**0.018**
Residual	4			38.1	
Total	11				
*(b) Bird sites: plant community composition*					
**Invasion Status**	1	2.59	**0.003**	16.4	**0.019**
**Region**	1	1.88	0.081 (MC)	21.3	**0.012**
**Site Pair (Region)**	6	3.03	**0.001**	37.1	
**Invasion Status × Region**	1	1.27	0.229	9.6	**0.001**
Residual	6			36.8	
Total	15				

### A.2 Bird functional groups

**TABLE A3:** Bird species and number of individuals observed in this study (*n* = 32 surveys), grouped by their diet, foraging strata, and scale (movement patterns) based on literature (Smyth et al. 2009, Tischler et al. 2013, Young and Schlesinger 2014, Garnett et al. 2015, Jordan et al. 2017, Gibson et al. 2022, Gorta et al. 2022). These functional groups were then combined to make two‐way diet × movement, strata × movement, and diet × strata functional groups for analysis.

Bird species	Scientific name	Number observed	Diet guild	Movement patterns	Foraging strata	Group taxon
Australian Magpie	*Gymnorhina tibicen*	2	Omnivore	Local‐landscape	Flexible	Artamidae
Australian Pipit	*Anthus australis*	8	Insectivore	Local‐landscape	Ground	Motacillidae
Black‐faced Cuckooshrike	*Coracina novaehollandiae*	12	Omnivore	Landscape	Flexible	Campephagidae
Black‐faced Woodswallow	*Artamus cinereus*	25	Insectivore	Local‐landscape	Flexible	Artamidae
Brown Songlark	*Megalurus cruralis*	1	Insectivore	Local‐landscape	Ground	Locustellidae
Budgerigar	*Melopsittacus undulatus*	10	Granivore	Landscape	Ground	Psittaciformes
Crested Bellbird	*Oreoica gutturalis*	6	Omnivore	Local	Flexible	Psophididae
Crested Pigeon	*Ocyphaps lophotes*	2	Granivore	Local	Ground	Columbiformes
Dusky Grasswren	*Amytornis purnelli*	13	Insectivore	Local	Ground	Maluridae
Galah	*Eolophus roseicapilla*	20	Granivore	Local	Flexible	Psittaciformes
Grey Shrikethrush	*Colluricincla harmonica*	7	Omnivore	Landscape	Flexible	Pachycephalidae
Grey‐fronted Honeyeater	*Ptilotula plumula plumula*	3	Omnivore	Landscape	Above ground	Meliphagidae
Grey‐headed Honeyeater	*Ptilotula keartlandi*	8	Nectar‐Frugivore	Local	Above ground	Meliphagidae
Hooded Robin	*Melanodryas cucullata westralensis*	2	Insectivore	Local	Flexible	Petrocidae
Inland Thornbill	*Acanthiza apicalis*	8	Insectivore	Local	Above ground	Acanthizidae
Mistletoebird	*Dicaeum hirundinaceum*	4	Nectar‐Frugivore	Landscape	Above ground	Dicaeidae
Nankeen Kestrel	*Falco cenchroides*	4	Carnivore	Local‐landscape	Above ground	Falconiformes
Peregrine Falcon	*Falco peregrinus*	1	Carnivore	Local‐landscape	Above ground	Falconiformes
Pied Butcherbird	*Cracticus nigrogularis*	4	Carnivore	Local	Flexible	Artamidae
Rainbow Bee‐eater	*Merops ornatus*	1	Insectivore	Landscape	Above ground	Coraciiformes
Red‐browed Pardalote	*Pardalotus rubricatus*	2	Insectivore	Local‐landscape	Above ground	Pardalotidae
Red‐capped Robin	*Petroica goodenovii*	2	Insectivore	Local‐landscape	Flexible	Petrocidae
Rufous Songlark	*Megalurus mathewsi*	1	Insectivore	Landscape	Ground	Locustellidae
Rufous Whistler	*Pachycephala rufiventris rufiventris*	10	Insectivore	Landscape	Flexible	Pachycephalidae
Singing Honeyeater	*Gavicalis virescens*	46	Omnivore	Local‐landscape	Above ground	Meliphagidae
Spiny‐cheeked Honeyeater	*Acanthagenys rufogularis*	26	Omnivore	Local‐landscape	Above ground	Meliphagidae
Torresian Crow	*Corvus orru*	10	Omnivore	Landscape	Flexible	Corvidae
Turquoise Fairywren	*Malurus splendens callainus*	1	Insectivore	Local	Flexible	Maluridae
Variegated Fairywren	*Malurus lamberti*	6	Insectivore	Local	Flexible	Maluridae
Wedge‐tailed Eagle	*Aquila audax*	2	Carnivore	Local‐landscape	Above ground	Accipitriformes
Western Bowerbird	*Chlamydera guttata*	5	Nectar‐Frugivore	Local	Flexible	Ptilonorhyn‐chidae
Western Gerygone	*Gerygone fusca mungi*	1	Insectivore	Landscape	Above ground	Acanthizidae
Whistling Kite	*Haliastur sphenurus*	1	Carnivore	Landscape	Above ground	Accipitriformes
White‐backed Swallow	*Cheramoeca leucosterna*	3	Insectivore	Local	Above ground	Hirundinidae
White‐fronted Honeyeater	*Purnella albifrons*	1	Nectar‐Frugivore	Landscape	Above ground	Meliphagidae
White‐winged Fairywren	*Malurus leucopterus*	9	Insectivore	Local	Flexible	Maluridae
Willie Wagtail	*Rhipidura leucophrys*	9	Insectivore	Local‐landscape	Flexible	Rhipiduridae
Yellow‐throated Miner	*Manorina flavigula*	27	Omnivore	Local	Above ground	Meliphagidae
Zebra Finch	*Taeniopygia guttata*	21	Granivore	Local‐landscape	Ground	Estrildidae

### A.3 Bird multivariate analysis

**TABLE A4:** Multivariate PERMANOVA and PERMDISP for (a) bird species community composition, (b) functional composition of the bird community delineated by diet × movement, (c) foraging strata × movement, and (d) diet × foraging strata, as a function of site Invasion Status × Region (fixed factors) and Site Pair (random factor) nested in region, to account for the paired‐plot design.

Predictor variable	df	Pseudo‐F	P(perm)	ECV	*PERM DISP p*
*(a) Bird species composition*					
**Invasion Status**	1	1.08	0.388	5.2	**0.007**
Region	1	2.14	0.055 (MC)	21.8	0.110
Site Pair (Region)	6	1.31	0.132	19.8	
Invasion Status × Region	1	0.86	0.603	−9.4	0.359
Residual	6			50.5	
Total	15				
*(b) Bird functional composition diet* × *movement*					
**Invasion Status**	1	0.96	0.490	−2.7	**0.007**
Region	1	2.13	0.065 (MC)	17.6	0.145
Site Pair (Region)	6	1.59	0.073	20.2	
Invasion Status × Region	1	1.12	0.386	6.4	0.410
Residual	6			37.0	
Total	15				
*(c) Bsird functional composition foraging strata* × *movement*					
**Invasion Status**	1	0.32	0.895	−10.3	**0.002**
Region	1	2.17	0.082 (MC)	16.5	0.096
Site Pair (Region)	6	1.47	0.132	17.2	
Invasion Status × Region	1	1.76	0.139	15.4	0.366
Residual	6			35.5	
Total	15				

Nonsignificant Invasion Status					
*(d) Bird functional composition foraging strata* × *diet*					
Invasion Status	1	1.44	0.219	8.0	0.146
Region	1	1.77	0.167 (MC)	13.5	0.658
Site Pair (Region)	6	1.61	0.058	18.9	
Invasion Status × Region	1	Negative		−17.5	0.568
Residual	6			34.4	
Total	15				

*Note:* All models used Type III Sums of squares and unrestricted permutations of raw data. The number of permutations to obtain *p* values was ≥ 998 for all predictors except region, where a Monte Carlo (MC) permutation was used to obtain the *p* value. ECV (estimate of components of variation) indicates relative importance of each predictor in the model and was calculated by sums of squared fixed or random effects, divided by degrees of freedom, and square root taken to represent dissimilarity units. PERMDISP used distance to centroid. Significant predictors are highlighted in bold for PERMANOVA or PERMDISP *v* < 0.05. Analysis used (a) bird mean abundance data, then (b), (c), (d) aggregated abundance into functional groups, and all matrices were square root transformed and used percentage difference (Bray–Curtis) resemblance. *N* = 16 sites.

**TABLE A5:** Bird species percent contribution to the first 70% of dissimilarity between native sites and invaded sites (SIMPER, based on species mean abundance, square root transformed), sorted by species with higher abundance in native sites (blue) and higher abundance in invaded sites (red), and then ranked by contribution to total dissimilarity (Contrib %). Diet, foraging strata, and movement functional groups also included.

Higher average abundance	Bird species	Contrib %	Diet	Foraging strata	Scale
Native	Spiny‐cheeked Honeyeater	6.25	Omnivore	Aboveground	Local‐landscape
Native	Black‐faced Woodswallow	5.96	Insectivore	Flexible	Local‐landscape
Native	Zebra Finch	5.39	Granivore	Ground	Local‐landscape
Native	Dusky Grasswren	4.73	Insectivore	Ground	Local
Native	Australian Pipit	3.49	Insectivore	Ground	Local‐landscape
Native	Rufous Whistler	3.44	Insectivore	Flexible	Landscape
Native	Grey Shrikethrush	3.42	Omnivore	Flexible	Landscape
Native	Galah	3.31	Granivore	Flexible	Local
Native	Grey‐headed Honeyeater	2.88	Nectar‐Frugivore	Aboveground	Local
Invaded	Singing Honeyeater	8.59	Omnivore	Aboveground	Local‐landscape
Invaded	Yellow‐throated Miner	5.79	Omnivore	Aboveground	Local
Invaded	Torresian Crow	4.19	Omnivore	Flexible	Landscape
Invaded	Willie Wagtail	3.89	Insectivore	Flexible	Local‐landscape
Invaded	Black‐faced Cuckooshrike	3.6	Omnivore	Flexible	Landscape
Invaded	White‐winged Fairywren	3.37	Insectivore	Flexible	Local
Invaded	Crested Bellbird	2.79	Omnivore	Flexible	Local

**TABLE A6:** SIMPER analysis for the significant difference between native and invaded bird community compositions (PERMDISP), when bird abundance was aggregated into two‐way functional groups of diet × movement. Groups Native & Invaded Average dissimilarity = 59.76/100, of square root (sq rt) abundance.

Two‐way functional group	Average native sq rt abundance	Average invaded sq rt abundance	Av Diss	Diss/SD	Contrib. %	Cumul.%	Sample size		
							Sites	Spp.	Obs.
Omnivore.local_landscape	1.01	1.6	8.3	1.4	13.9	13.9	13	3	74
Insectivore.local_landscape	1.01	0.81	7.3	1.2	12.2	26.0	10	6	47
Insectivore.local	0.93	0.81	6.9	1.2	11.5	37.5	11	7	42
Omnivore.local	0.54	0.76	6.3	1.1	10.6	48.1	8	2	33
Omnivore.landscape	0.53	1.12	5.1	1.1	8.5	56.6	13	4	33
Granivore.local_landscape	0.45	0.21	4.8	0.6	8.0	64.6	4	1	21
Granivore.local	0.48	0.09	4.1	0.5	6.9	71.5	3	2	22
Insectivore.landscape	0.55	0.18	4.0	0.9	6.7	78.2	6	4	13
Nectar_Frugivore.local	0.31	0.32	3.5	0.7	5.8	84.0	4	2	12
Carnivore.local_landscape	0.21	0.3	2.9	0.8	4.8	88.8	5	3	7
Carnivore.local	0.09	0.27	2.3	0.7	3.9	92.8	4	1	4
Granivore.landscape	0	0.28	1.9	0.4	3.2	96.0	1	1	10
Nectar‐Frugivore.landscape	0.27	0	1.8	0.5	3.0	99.0	2	2	5
Carnivore.landscape	0.09	0	0.6	0.4	1.0	100.0	1	1	1

Abbreviations: Av Diss = average dissimilarity (out of 100 units); Contrib. % = species contribution to total community dissimilarity; Cumul. % = cumulative contribution; Diss/SD = variation in dissimilarity; Obs. = number of observations for each functional group; Sites = number of sites each two‐way functional groups occurred in; Spp. = number of species in each functional group.

**TABLE A7:** SIMPER analysis for the significant difference between bird community composition (PERMDISP) at native and invaded sites, when bird abundance was aggregated into two‐way functional groups of foraging strata × movement. Groups Native & Invaded Average dissimilarity = 54.96/100, of square root (sq rt) abundance.

Two‐way functional group	Average native sq rt abundance	Average invaded sq rt abundance	Av Diss	Diss/SD	Contrib. %	Cumul.%	Sample size		
							Sites	Spp.	Obs.
Flexible.local	0.93	0.77	8.9	1.1	16.2	16.2	9	8	53
Above ground.local_landscape	1.14	1.66	8.5	1.4	15.5	31.6	14	6	81
Above ground.local	0.69	0.75	7.5	1.0	13.6	45.2	6	4	45
Flexible.local_landscape	0.87	0.78	6.8	1.3	12.4	57.7	10	4	38
Ground.local_landscape	0.73	0.35	6.8	0.8	12.3	70.0	7	3	30
Ground.local	0.41	0.3	5.2	0.8	9.5	79.5	5	2	15
Flexible.landscape	0.81	1.05	5.2	1.1	9.4	88.8	14	4	35
Above ground.landscape	0.32	0.24	3.7	0.8	6.7	95.6	4	6	16
Ground.landscape	0.09	0.28	2.4	0.5	4.4	100.0	5	3	11

Abbreviations: Av Diss = average dissimilarity (out of 100 units); Contrib. % = species contribution to total community dissimilarity; Cumul. % = cumulative contribution; Diss/SD = variation in dissimilarity; Obs. = number of observations for each functional group; Sites = number of sites each two‐way functional groups occurred in; Spp. = number of species in each functional group.

### A.4 Reptile functional groups

**TABLE A8:** Reptile capture rates, functional groups, and literature sources for defining diet and habitat functional groups. Body size snout vent length (unless indicated as TL = total length) taken from Wilson and Swan (2013). Full literature citations located at the end of the Appendix A1.

Reptile name	Common name	Individuals captured	Body size^#^	Body size class	Diet functional group assigned in this study	Habitat use functional group assigned in this study	Diet literature	Habitat use literature
*Anilios endoterus*	Desert Blind Snake	1	376^TL^	350_800	Invertebrate specialist	Subterranean	Wilson and Swan 2003	Wilson and Swan 2003
*Brachyurophis semifasciatus*	Southern Shovel‐nose Snake	1	353^TL^	350_800	Vertebrate specialist	Vegetated‐open	Goodyear and Pianka 2008	Goodyear and Pianka 2008
*Carlia triacantha*	Desert Rainbow Skink	1	53	30_80	Invertebrate generalist	Vegetated	Manicom and Schwarzkopf 2011 for congeneric *Carlia*	Manicom and Schwarzkopf 2011 for congeneric *Carlia*
*Ctenophorus isolepis*	Military Dragon	21	70	30_80	Invertebrate specialist	Vegetated	Pianka 1986, Daly et al. 2008	Daly et al. 2007, Daly et al. 2008
*Ctenophorus nuchalis*	Central Netted Dragon	2	115	90_140	Invertebrate generalist	Open	Pianka 1986, Daly et al. 2008	Daly et al. 2007, Daly et al. 2008
*Ctenotus calurus*	Blue‐Tailed Ctenotus	1	49	30_80	Invertebrate generalist	Vegetated‐Open	Letnic et al. 2004, Goodyear and Pianka 2011	Pianka 1986
*Ctenotus leonhardii*	Common Desert Ctenotus	6	78	30_80	Invertebrate generalist	Vegetated	James 1991	Pianka 1986
*Ctenotus pantherinus*	Leopard Ctenotus	2	114	90_140	Invertebrate generalist	Vegetated	James 1991, Letnic et al. 2004, Goodyear and Pianka 2011	Pianka 1986
*Ctenotus quattuordecimlineatus*	Many‐lined Ctenotus	1	70	30_80	Invertebrate generalist	Vegetated	James 1991, Letnic et al. 2004, Goodyear and Pianka 2011	Pianka 1986
*Ctenotus schomburgkii*	Common Sandplain Ctenotus	3	52	30_80	Invertebrate specialist	Vegetated‐Open	Pianka 1986	Pianka 1986
*Ctenotus sp*.	Ctenotus	1	65	30_80	Invertebrate generalist	Vegetated	Pianka 1986	Pianka 1986
*Cyclodomorphus melanops*	Spinifex Slender Bluetongue	1	132	90_140	Invertebrate generalist	Vegetated	Pianka 2011	Pianka 2011
*Delma borea*	Rusty‐topped Delma	3	95	90_140	Invertebrate generalist	Vegetated	Patchell and Shine 1986, Pianka 1986, Letnic et al. 2004 for congeneric *Delma*	Pianka 1986
*Demansia sp*.	Whip snake	1	800^TL^	350_800	Vertebrate generalist	Vegetated‐Open	Wilson and Swan 2013	Nankivell et al. 2023
*Diplodactylus conspicillatus*	Variable Fat‐tailed Gecko	4	65	30_80	Invertebrate specialist	Vegetated‐Open	Pianka 1986, Letnic et al. 2004	Pianka 1986, Letnic et al. 2004
*Gehyra purpurascens*	Robust Tree Gehyra	3	64	30_80	Invertebrate generalist	Vegetated‐Arboreal	Pianka 1986, Letnic et al. 2004 for congeneric *Gehyra*	Pianka 1986, Letnic et al. 2004 for congeneric *Gehyra*
*Gehyra variegata*	Western Tree Gehyra	1	54	30_80	Invertebrate generalist	Vegetated‐Arboreal	Pianka 1986, Whithers et al. 2000	Grimm‐Seyfarth et al. 2018
*Gehyra versicolor*	Eastern Tree Gehrya	1	54	30_80	Invertebrate generalist	Vegetated‐Arboreal	Letnic et al. 2004	Riedel et al. 2020
*Gowidon longirostris*	Long‐Nosed Dragon	2	114	90_140	Invertebrate generalist	Vegetated‐Arboreal	Pianka 1986, Letnic et al. 2004	Pianka 1986
*Heteronotia binoei*	Bynoe's Gecko	1	54	30_80	Invertebrate generalist	Vegetated‐Arboreal	Pianka 1986, Letnic et al. 2004	Riedel et al. 2020
*Lerista labialis*	Eastern Two‐toed Slider	50	60	30_80	Invertebrate specialist	Subterranean	Greenville and Dickman 2005	Greenville and Dickman 2005
*Lialis burtonis*	Burton's Legless Lizard	2	290	220_300	Vertebrate generalist	Open	Patchell and Shine 1986, Pianka 1986	Riedel et al. 2020
*Lucasium stenodactylum*	Gecko	2	57	30_80	Invertebrate generalist	Open	Pianka 1986, Letnic et al. 2004	Riedel et al. 2020
*Menetia greyii*	Common Dwarf Skink	1	38	30_80	Invertebrate generalist	Vegetated	Pianka 1986, Letnic et al. 2004	Pianka 1986, Letnic et al. 2004
*Moloch horridus*	Thorny Devil	2	110	90_140	Invertebrate specialist	Vegetated	Pianka 1986, Letnic et al. 2004	Pianka 1986
*Nephrurus levis*	Common Knob‐tailed Gecko	3	102	90_140	Invertebrate generalist	Vegetated‐Open	Pianka 1986, Letnic et al. 2004	Pianka 1986, Riedel et al. 2020
*Pseudonaja mengdeni*	Western Brown Snake	1	800^TL^	350_800	Vertebrate generalist	Vegetated‐Open	Wilson and Swan 2013	Wilson and Swan 2013
*Pygopus nigriceps*	Western Hooded Scaly‐foot	1	227	220_300	Invertebrate generalist	Open	Pianka 1986	Pianka 1986
*Rhynchoedura ornata*	Western Beaked Gecko	4	57	30_80	Invertebrate specialist	Open	Letnic et al. 2004	Pianka 1986
*Tympanocryptis centralis*	Centralian Earless Dragon	5	57	30_80	Invertebrate generalist	Vegetated‐Open	Starr and Leung 2010 for congeneric *Tympanocryptis*	Stevens et al. 2010 for congeneric *Tympanocryptis*, Chaplin 2018

### A.5 Reptile multivariate analysis

**TABLE A9:** Multivariate PERMANOVA and PERMDISP for (a) reptile species community composition, (b) functional composition of the reptile community delineated by habitat use × body size, (c) diet × habitat use, and (d) diet × body size, as a function of site Invasion Status × Region (fixed factors) and Site Pair (random factor) nested in region, to account for the paired‐plot design.

Predictor variable	df	Pseudo‐F	P(perm)	ECV	PERM DISP *p*
*(a) Reptile species composition*					
Invasion Status	1	1.31	0.239	10.3	0.948
Region	1	1.19	0.347 (MC)	12.3	0.050
**Site Pair (Region)**	4	2.39	**0.001**	37.5	
Invasion Status × Region	1	1.18	0.316	10.9	0.444
Residual	4			45.0	
Total	11				
*(b) Reptile functional composition habitat use* × *body size*					
**Invasion Status**	1	3.69	**0.020**	20.9	0.808
Region	1	1.95	0.181 (MC)	21.9	0.070
**Site Pair (Region)**	4	3.08	**0.010**	31.9	
Invasion Status × Region	1	2.90	0.053	24.9	0.38
Residual	4			31.3	
Total	11				

Nonsignificant invasion status					
*(c) Reptile functional composition diet* × *habitat use*					
Invasion Status	1	1.98	0.122	13.1	0.950
Region	1	1.20	0.380(MC)	11.1	0.080
**Site Pair (Region)**	4	3.44	**0.004**	35.8	
Invasion Status × Region	1	1.18	0.346	7.8	0.213
Residual	4			32.4	
Total	11				
*(d) Reptile functional composition diet* × *body size*					
Invasion Status	1	1.28	0.344	8.6	0.215
Region	1	0.65	0.613(MC)	−9.4	0.265
Site Pair (Region)	4	0.94	0.568	−6.8	
Invasion Status × Region	1	1.42	0.256	15.0	0.082
Residual	4			40.1	
Total	11				

*Note:* All models used Type III sums of squares and unrestricted permutations of raw data. Number of permutations to obtain *p* values was ≥ 998 for all predictors except region, where a Monte Carlo (MC) permutation was used to obtain the *p* value. ECV (estimate of components of variation) indicates relative importance of each predictor in the model and was calculated by sums of squared fixed or random effects, divided by degrees of freedom, and square root taken to represent dissimilarity units. PERMDISP used distance to centroid. Significant predictors are highlighted in bold for PERMANOVA or PERMDISP *p* < 0.05. Analysis used (a) reptile abundance data, and (b), (c), (d) aggregated abundances into functional groups, and all matrices were square root transformed and used percentage‐difference (Bray–Curtis) resemblance. *N* = 12 sites.

**TABLE A10:** SIMPER analysis for the significant difference between native and invaded reptile community compositions (PERMANOVA), when reptile abundance was aggregated into two‐way functional groups of habitat use × body size. Groups Native & Invaded Average dissimilarity = 67.79/100, of square root (sq rt) abundance.

Two‐way functional group	Average native sq rt abundance	Average invaded sq rt abundance	Av Diss	Diss/SD	Contrib.%	Cumul.%	Sample size		
							Sites	Spp.	Obs.
Subterrenean.30_80	1.39	1.65	14.0	1.1	20.6	20.6	7	1	50
Vegetated_Ground.30_80	1.39	1.25	10.9	1.2	16.1	36.7	9	6	31
Vegetated_Ground.90_140	0.24	0.79	8.9	1.0	13.1	49.7	5	4	8
Vegetated_Open.30_80	0.8	0.5	8.6	0.7	12.7	62.4	6	4	13
Open_Ground.30_80	0.5	0.17	6.1	0.6	9.0	71.4	3	2	6
Vegetated_Aboreal.30_80	0.33	0.57	5.0	1.0	7.4	78.8	5	4	6
Open_Ground.220_300	0.5	0	4.7	0.9	6.9	85.7	3	2	3
Open_Ground.90_140	0.33	0	2.5	0.7	3.6	89.3	2	1	2
Vegetated_Open.350_800	0	0.29	2.2	0.4	3.2	92.5	1	3	3
Vegetated_Open.90_140	0.29	0	2.1	0.4	3.1	95.6	1	1	3
Vegetated_Aboreal.90_140	0	0.24	1.8	0.4	2.6	98.2	1	1	2
Subterrenean.350_800	0	0.17	1.2	0.4	1.8	100.0	1	1	1

Abbreviation: Av Diss = average dissimilarity (out of 100 units), Contrib. % = species contribution to total community dissimilarity, Cumul. % = cumulative contribution, Diss/SD = variation in dissimilarity, Obs. = number of observations for each functional group, Sites = number of sites each two‐way functional groups occurred in, Spp. = number of species in each functional group.

### A.6 Ant multivariate analysis and functional groups

**TABLE A11:** Multivariate PERMANOVA and PERMDISP for (a) ant taxa community composition (genera) and (b) functional composition of the ant community delineated by ant functional groups (Anderson 1995), as a function of site invasion status × region (fixed factors) and site pair (random factor) nested in region to account for the paired‐plot design.

Predictor variable	df	Pseudo‐F	P(perm)	ECV	PERM DISP *p*
*(a) Ant taxa composition*					
Invasion Status	1	0.84	0.599	−4.1	0.657
Region	1	1.26	0.296 (MC)	6.6	0.074
Site Pair (Region)	4	1.64	0.071	14.0	
Invasion Status × Region	1	1.17	0.372	5.9	0.178
Residual	4			24.8	
Total	11				
*(b) Ant functional composition*					
**Invasion Status**	1	5.34	**0.005**	10.5	0.785
Region	1	0.80	0.509 (MC)	−3.8	0.338
Site Pair (Region)	4	2.78	0.018	11.7	
**Invasion Status × Region**	1	3.68	**0.020**	11.7	0.805
Residual	4			12.4	
Total	11				

**TABLE A12:** Ant SIMPER analysis, functional groups, and sample size, organised by largest contributors to dissimilarity and higher abundance in native sites (blue), through to largest contributors to dissimilarity and higher abundance in invaded sites (red), and characteristics of functional groups (Andersen 1995) across three functional axes: Temperature tolerance from open to more shaded habitat requirements; ant hierarchy, from solitary and poorly competitive ants through to competitive and dominant groups than can form large aggregations; diet, from lower trophic specialists through to higher trophic levels.

Functional groups and genera (Andersen 1995)	Average fourth root abundance		Contrib%	Temp. toler.	Hierarchy (aggregation size)	Diet	Total captures	Number of sites
	Native	Invaded						
General Myrmicinae (GM) *Crematogaster, Pheidole*	1.88	0.85	21.27	broad	Competitive but avoid dominant	**Includes specialist seed harvesters**	167	9
Hot Climate Specialists (HCS) *Melophorus, Meranoplus, Monomorium* ^a^	3.08	2.58	13.72	**hot**	Competitive but avoid dominant	**Includes specialist seed harvesters**	1058	12
Dominant Dolichoderinae (DD) *Iridomyrmex*	3.3	2.71	13.68	**hot**	Dominant	Omnivores—plant dominant	1308	12
Subordinate Camponoti (SC) *Calomyrmex, Camponotus, Polyrachis*	1.48	1.14	2.84	broad	Competitive but avoid dominant	Omnivores—plant dominant	68	11
Cold Climate Specialists (CCS) *Stigmacros*	0.17	0	2.77	cooler	Competitive but avoid dominant	Omnivores—animal dominant	1	1
Specialist Predators (SP) *Cerapachys, Leptogenys, Odontomachus*	0.36	0.81	11.5	broad	**Poor competitors**	Predators	12	6
Cryptic species (CRY) *Hypoponera, Pseudoneoponera, Solenopsis*	0.28	0.72	12.61	cooler	**Poor competitors**	Omnivores—animal dominant	14	5
Oppourtunists (OPP) *Doleromyrma, Rhytidoponera, Tapinoma, Tetramorium*	1.25	1.75	13.85	broad	**Poor competitors**	Omnivores—animal dominant	127	10

*Note*: Bold indicates which groups within each functional axes were predicted to be most impacted by grass invasion based on our conceptual model. Capture rates and site occurrence out of *n* = 12 sites.

Abbreviation: Temp. toler. = Temperature tolerance.

^a^ *Monomorium* was included in hot climate specialists (HCS) as the majority of the *Monomorium* specimens were in the *Monomorium rothsteini* group, which is classified as HCS in this region (Bonney et al. 2016), whereas some other *Monomorium* species groups are considered part of general myrmicinae.

### A.7 Taxa richness and abundance model outputs

**TABLE A13:** Model parameters and outputs for total abundance and species diversity for three taxonomic groups (birds, reptiles, and ants) as a function of site Invasion Status and Site Pair (random factor). Model outputs are from the most parsimonious model for each response variable using lowest AICc, or if the top model showed poor diagnostics, the next model was chosen within Delta AICc < 3. Significant effects are highlighted in bold. lmer = linear mixed model, glm = generalised linear model, lm = linear model. *n* = 16 sites for birds and *n* = 12 for reptiles and ants.

Effect	Predictor	Estimate	se	Statistic	2.5% CI	97.5% CI	*p* (S)	*p* (LRT)
**Bird abundance**								
lmer: bird abundance ~ Invasion Status + (1|Site Pair) Pseudo‐*R*² (fixed effects) = 0.009 Pseudo‐*R*² (total) = 0.142								
*fixed*	(Intercept)	10.69	1.95	5.47	6.86	14.51	< 0.001	
*fixed*	Invasion Status:Invaded	−1.00	2.57	−0.39	−6.04	4.04	0.707	0.699
*rand*	Site Pair (sd_Intercept)	2.02						
*rand*	Residual (sd_Observation)	5.14						0.703
**Bird species diversity**								
lmer: bird Shannon ENS ~ Invasion Status + (1|Site Pair) Pseudo‐*R*² (fixed effects) = 0.042 Pseudo‐*R*² (total) = 0.181								
*fixed*	(Intercept)	4.91	0.93	5.26	3.08	6.74	< 0.001	
*fixed*	Invasion Status:Invaded	1.07	1.22	0.87	−1.33	3.46	0.408	0.393
*rand*	Site Pair (sd_Intercept)	1.01						
*rand*	Residual (sd_Observation)	2.44						0.680
**Reptile abundance**								
lmer: reptile abundance ~ Invasion Status + (1|Site Pair) Pseudo‐*R*² (fixed effects) = 0.008 Pseudo‐*R*² (total) = 0.619								
*fixed*	(Intercept)	11.17	2.31	4.82	6.63	15.70	0.001	
*fixed*	Invasion Status:Invaded	−1.00	2.03	−0.49	−4.97	2.97	0.639	0.625
*rand*	Site Pair (sd_Intercept)	4.45						
*rand*	Residual (sd_Observation)	3.51						0.090
**Reptile species diversity**								
lmer: reptile Shannon ENS ~ Invasion Status + (1|Site Pair) Pseudo‐*R*² (fixed effects) = 0.000 Pseudo‐*R*² (total) = 0.048								
*fixed*	(Intercept)	3.67	0.65	5.68	2.40	4.93	< 0.001	
*fixed*	Invasion Status:Invaded	−0.01	0.89	−0.01	−1.76	1.73	0.989	0.989
*rand*	Site Pair (sd_Intercept)	0.35						
*rand*	Residual (sd_Observation)	1.54						0.906
effect	predictor	estimate	se	statistic	2.5% CI	97.5% CI	*p* (S)	*p* value (LRT)
**Ant abundance**								
glm (negative binomial, log link): ant abundance ~ Invasion Status								
	Predictor	exp (estimate)	exp (se)	Statistic	exp (2.5% CI)	exp (97.5% CI)	*p* (W)	*p* (LRT)
*fixed*	(Intercept)	303.17	0.18	32.49	218.68	436.73	< 0.001	
*fixed*	**Invasion Status:Invaded**	**0.41**	**0.25**	−**3.53**	**0.25**	**0.68**	**< 0.001**	
**Ant taxa diversity**								
lm: Ant Shannon ENS ~ Invasion Status *R*² = 0.24 Adj. *R*² = 0.17								
	Predictor	Estimate	se	Statistic	2.5% CI	97.5% CI	*p*	*p* (LRT)
*fixed*	(Intercept)	2.69	0.36	7.48	1.89	3.50	< 0.001	
*fixed*	Invasion Status:Invaded	0.91	0.51	1.80	−0.22	2.05	0.103	0.103

*Note:* Significant effects are when the 95% confidence interval does not overlap zero for lm or lmer, or one for glm (exp (0) = 1), then considering the *p* value derived from likelihood ratio tests (LRT), Satterthwaite degrees of freedom (S) or Wald's statistic (W). When modelling ant abundance and diversity, Site Pair (random effect) had near zero variance and was contributing to a singular fit, and thus, we removed Site Pair, which improved model diagnostics and lowered the AICc. Models including region were tested but were not the most parsimonious model.

Abbreviations: CI = confidence interval; ENS used effective number of species = exp(shannon diversity index H’); rand = random; se = standard error.
